# A Three-Dimensional Model of the Yeast Transmembrane Sensor Wsc1 Obtained by SMA-Based Detergent-Free Purification and Transmission Electron Microscopy

**DOI:** 10.3390/jof7020118

**Published:** 2021-02-05

**Authors:** Natalia Voskoboynikova, Maria Karlova, Rainer Kurre, Armen Y. Mulkidjanian, Konstantin V. Shaitan, Olga S. Sokolova, Heinz-Jürgen Steinhoff, Jürgen J. Heinisch

**Affiliations:** 1 Faculty of Physics, University of Osnabrück, Barbarastrasse 7, D-49076 Osnabrück, Germany; armen.mulkidjanian@uni-osnabrueck.de (A.Y.M.); hsteinho@uni-osnabrueck.de (H.-J.S.); 2 Department of Bioengineering, Faculty of Biology, M. V. Lomonosov Moscow State University, 119234 Moscow, Russia; mkarlova@yandex.ru (M.K.); shaytan49@yandex.ru (K.V.S.); sokolova@mail.bio.msu.ru (O.S.S.); 3 Center of Cellular Nanoanalytics, Integrated Bioimaging Facility iBiOs, Faculty of Biology/Chemistry, University of Osnabrück, Barbarastrasse 11, D-49076 Osnabrück, Germany; rainer.kurre@uos.de; 4 Faculty of Bioengineering and Bioinformatics, Belozersky Institute of Physico-Chemical Biology, M. V. Lomonosov Moscow State University, 119234 Moscow, Russia; 5 N.N. Semenov Federal Research Center for Chemical Physics, Russian Academy of Sciences, 119991 Moscow, Russia; 6 Biology Department, Shenzhen MSU-BIT University, Shenzhen 518172, China; 7 Department of Genetics, Faculty of Biology/Chemistry, University of Osnabrück, Barbarastrasse 11, D-49076 Osnabrück, Germany

**Keywords:** Wsc1, membrane sensor, SMALP, detergent-free extraction, fluorescence correlation spectroscopy, transmission electron microscopy, 3D reconstruction

## Abstract

The cell wall sensor Wsc1 belongs to a small family of transmembrane proteins, which are crucial to sustain cell integrity in yeast and other fungi. Wsc1 acts as a mechanosensor of the cell wall integrity (CWI) signal transduction pathway which responds to external stresses. Here we report on the purification of Wsc1 by its trapping in water-soluble polymer-stabilized lipid nanoparticles, obtained with an amphipathic styrene-maleic acid (SMA) copolymer. The latter was employed to transfer tagged sensors from their native yeast membranes into SMA/lipid particles (SMALPs), which allows their purification in a functional state, i.e., avoiding denaturation. The SMALPs composition was characterized by fluorescence correlation spectroscopy, followed by two-dimensional image acquisition from single particle transmission electron microscopy to build a three-dimensional model of the sensor. The latter confirms that Wsc1 consists of a large extracellular domain connected to a smaller intracellular part by a single transmembrane domain, which is embedded within the hydrophobic moiety of the lipid bilayer. The successful extraction of a sensor from the yeast plasma membrane by a detergent-free procedure into a native-like membrane environment provides new prospects for in vitro structural and functional studies of yeast plasma proteins which are likely to be applicable to other fungi, including plant and human pathogens.

## 1. Introduction

Fungal cell walls are essential structures that serve as a first barrier against adverse environmental conditions and ensure form and stability of yeast cells and hyphae in filamentous fungi. As such, they are a prime choice for the development of antifungal drugs and of utmost importance in medicine and agriculture [1]. In this context, cell wall biogenesis and its regulation has been extensively studied in the yeast *Saccharomyces cerevisiae* [2]. In short, cell surface stress is detected by a small family of membrane spanning sensors, which, mediated by the yeast protein kinase C (Pkc1), signal to a mitogen-activated protein kinase (MAPK) cascade to nuclear transcription factors that promote cell wall reconstruction (reviewed in [3]). Taken together, the term cell wall integrity (CWI) pathway has been coined for this signal transduction cascade in yeast and has been adopted for homologous systems in other fungi [4,5]. Amongst the five sensors of *S. cerevisiae*, Wsc1 has best been studied both by genetic and biophysical methods (reviewed in [6]). Atomic-force microscopy (AFM) experiments suggest that individual Wsc1 proteins act as mechanosensors in the form of a nanospring [7]. AFM studies also showed that the clustering of sensors in the plasma membrane in response to cell surface stress is controlled by the cysteine-rich extracellular domain (CRD, also known as the Wsc-domain; [3,5]). Quantitative live cell fluorescence microscopy showed that, in cells growing under standard conditions, Wsc1 is localized in specific microdomains of the plasma membrane, termed Membrane Compartment carrying Wsc1 (MCW) [8].

In all studies performed so far, Wsc1 functions were assessed either in living yeast cells [7,9,10] or in crude cell extracts [5,11,12]. No studies on the purification of the sensor, much less in a native membrane environment, were available, until now. The routine isolation and purification of membrane proteins in most studies involves the use of detergents. While such approaches allow structural assessments of membrane proteins (MPs) in solution, they may negatively affect their stability and activity of the protein in question, thus leading to modifications and even complete inactivation [13].

To overcome these technical barriers, novel membrane mimetic systems for MPs on the nanometer scale have been developed, which avoid the use of detergents [14]. Amphipathic styrene-maleic acid (SMA) and SMA-related copolymers enable the direct, detergent-free extraction of membrane proteins from both artificial membranes with a given composition of lipids, and natural lipid bilayers, providing stable membrane patches with incorporated MPs (reviewed in [11,15,16,17,18]). The SMA copolymer-driven lipid solubilization also has the advantage of being non-selective with regard to the lipid-type of the donor membrane [19,20]. SMA-encased lipoprotein particles comprise lipid/protein cores surrounded by a SMA copolymer belt and usually have diameters of approximately 10 nm, depending on the preparation routine [21]. The SMA copolymer with a 3:1 styrene-to-maleic acid molar ratio (3:1 SMA) has been shown to extract both α-helical bundle proteins, such as bacteriorhodopsin [14,22], and β-barrel proteins, such as lipid A palmitoyltransferase PagP [14], from 1,2-dimyristoyl-sn-glycero-3-phosphocholine (DMPC) liposomes. The non-selective nature of the method was also demonstrated by the extraction of transmembrane protein complexes from the archaeal photoreceptor–transducer complex (*Np*SRII/*Np*HtrII) into a 3:1 SMA copolymer, with a photoreceptor: transducer stoichiometry of 2:2, from *Escherichia coli* polar lipid liposomes [23]. Detergent-free solubilization of MPs by SMA copolymers has been demonstrated for cellular membranes from yeast [24,25,26,27,28] and mammalian cells [29].

An extensive set of biophysical methods is compatible with the SMA lipid particle (SMALP) system [30]. Thus, the small size and single-particle character of SMALPs facilitated the use of solution-based structural investigation by electron microscopy (EM; [31,32,33]).

In this work, we applied the 3:1 SMA copolymer to isolate Wsc1-GFP (green fluorescent protein) fusions into SMALPs directly from native yeast membranes. We purified Wsc1-GFP-containing SMALPs by affinity chromatography and characterized SMALP preparations using dynamic light scattering (DLS), fluorescence correlation spectroscopy (FCS) and single particle transmission electron microscopy (TEM). This serves as a proof-of-principle for further structural and functional studies of Wsc1 and other fungal MPs in vitro.

## 2. Materials and Methods

### 2.1. Media, Growth Conditions, Strains and Genetic Manipulations

Growth media and genetic manipulations followed previously described standard protocols [34]. The *S. cerevisiae* strain HOD356-1A (*MATa ura3-52 leu2-3,112 his3-11,15 WSC1-GFP-TEV-3xHA::SpHIS5*) was obtained in this work as a source of the Wsc1 sensor. It was constructed in the background of the HD56-5A strain by homologous recombination with PCR-generated tags at the native *WSC1* locus, based on constructs used in previous works [7,8,10]. Note that in addition to the green fluorescent protein (GFP) used for detection, the encoded fusion protein carries a recognition sequence for the TEV protease and a triple hemagglutinin tag (3 × HA) to facilitate purification. Details on the construction and the sequence of the genomic construct are available upon request.

The *WSC1* gene was expressed under the control of its native promoter. Yeast cells were grown to late logarithmic phase (~5 × 10^7^ cells/mL) in 50 mL of rich medium (1% *w/v* yeast extract, 2% *w/v* Bacto peptone; Difco Laboratories Inc., Detroit, MI, USA) with 2% *w/v* glucose as a carbon source (YEPD) at 30 °C with constant rotary shaking at 180 rpm.

### 2.2. Spheroplast Preparation

Yeast cells were harvested from 10 mL aliquots of the overnight culture by centrifugation to obtain approximately130 mg of wet cells washed once with 3 mL of sterile water and transferred to an Eppendorf tube. The wet cell pellet after centrifugation was resuspended in 0.5 mL of spheroplast buffer (0.9 M sorbitol, 0.1 M EDTA, 20 mM dithiothreitol). To digest the cell wall glucans, 2 µL Zymolyase 20T (an enzyme preparation from a submerged culture of *Arthrobacter luteus* provided by MP Biomedicals, Eschwege/Germany; applied from a stock solution of 10 mg/mL in sterile water and stored at −20 °C) were added and the suspension was incubated for 30 min at 37 °C. After centrifugation (13,500 rpm; 1 min; at room temperature), pellets containing the spheroplasts were either processed immediately or flash frozen and kept at −80 °C until further use.

### 2.3. Cell Lysis and Fractionation

Spheroplasts were washed twice and resuspended at 20 mg/mL of wet cells in buffer A: 10 mM Tris-HCl, pH 8.0, 150 mM NaCl supplemented with one tablet per 50 mL of protease inhibitor cocktail (Sigma-Aldrich, Merck KGaA, Darmstadt/Germany). The cell suspension was sonicated on ice (Branson Ultrasonic Corporation, USA; duty cycle 20; output control 2; 5 × 1 min with 1 min break in between). After centrifugation (134,000× *g*; 20 min; 4 °C), the resulting pellet was resuspended at 80 mg/mL of wet cells in buffer A and directly used for protein extraction by SMA or flash frozen and kept at −80 °C until further use.

### 2.4. Preparation of Styrene-Maleic Acid Copolymer Solution

The styrene maleic acid (SMA) copolymer with a styrene-to-maleic acid molar ratio of 3:1 (MW 9500 Da, supplied as an aqueous sodium salt solution SMA 3000 HNa) was kindly provided as a gift by Cray Valley (Exton; PA; USA). A 5% (*w/v*) solution of SMA, which was extensively dialyzed against buffer A, was used for the membrane solubilization.

### 2.5. Membrane Solubilization by SMA

A 5% (*w/v*) solution of SMA copolymer in buffer A was added dropwise to the membrane suspension to get a final cell-to-SMA weight ratio of 1:2.5. The suspension was incubated for 30 min at RT and then for 16 hrs at 4 °C. All incubations were performed with gentle shaking. The suspension was then centrifuged for 20 min at 134,000× *g* at 4 °C. Before the affinity purification, aliquots of the supernatant were taken for Dynamic Light Scattering and Fluorescence Correlation Spectroscopy analyses. The pellet and the supernatant were also analyzed by sodium dodecyl sulfate/polyacrylamide gel electrophoresis (SDS-PAGE) and immunoblotting. The supernatant was subsequently purified on affinity resin.

### 2.6. Affinity Chromatography

Anti-HA (hemagglutinin) agarose (Sigma-Aldrich, Munich, Germany) was pre-equilibrated with PBS buffer. SMA-solubilized membranes were added to the resin and incubated overnight at 4 °C with gentle mixing. The resin was then pelleted by brief centrifugation, and the supernatant (column flow through) was discarded. The resin was washed with 40 column volumes of PBS buffer and protein was eluted with four column volumes of the same buffer, supplemented with 100 µg/mL HA peptide (Sigma-Aldrich, Munich, Germany).

### 2.7. Electrophoresis and Immunoblot

All stages of cell fractionation, SMA solubilization and affinity purification were applied to 12% SDS-PAGE. For immunodetection proteins from gels were transferred onto a 0.2 µm nitrocellulose membrane (GE Healthcare, Chalfont St. Giles, UK), blocked with 5% non-fat milk and probed with rabbit polyclonal HA-antiserum (Abcam, Cambridge, UK) as primary antibodies. The secondary antibodies were anti-rabbit (H+L) HRP-conjugated (BioRad, Hercules, CA, USA). Chemiluminescent signals were developed with ECL SuperSignal West Pico substrate (Thermo, Bartlesville, OK, USA) and registered on a ChemiDoc XRS+ imager with ImageLab software (BioRad, Hercules, CA, USA).

### 2.8. Dynamic Light Scattering

Dynamic light scattering (DLS) measurements were performed on a Zetasizer Nano ZS (Malvern Instruments, Worcestershire, UK) at 550 nm and 25 °C. Data represent the average of three sets of 14 runs of 10 s each. The particle size distribution was obtained by using the ZETASIZER software package v7.02. under the assumption that the analyzed particles would have a spherical shape.

### 2.9. Fluorescence Correlation Spectroscopy

Fluorescence Correlation Spectroscopy (FCS) was conducted on a confocal laser scanning microscope (FluoView 1000, Olympus, Tokyo, Japan) equipped with a FLIM/FCS upgrade kit (Picoquant, Berlin, Germany). For excitation of GFP, a picosecond pulsed 485 nm laser diode (LDH-D-C-485, Picoquant) at a repetition rate of 20–40 MHz was used. Fluorescence was detected by a single photon avalanche detector (Picoquant) using a bandpass filter from 500–550 nm (BrightLine HC 525/50, Semrock, Tübingen, Germany). All measurements were performed in a 100 µL droplet placed on a high-precision coverslip at room temperature (24–26 °C). Exact temperature at sample position was determined for each measurement. Fluorescence was collected with a 60× water immersion objective (UPLSAPO 60×, NA 1.2, Olympus) at 20 µm above coverslip inside the droplet. Autocorrelation functions (ACF) were analyzed by the software SymPhoTime 64 (v2.5, Picoquant). This software allowed for correction of background and afterpulsing artefacts using fluorescence lifetime information. A diffusion model assuming a single population of particles showing three-dimensional free diffusion and considering triplet state blinking of GFP was fitted to the resulting ACFs [35]. FCS fitting requires the determination of the effective confocal volume, which can be calibrated with a dye solution with known diffusion constant measured at exact same acquisition conditions. We used a 1 nM fluorescein solution with a diffusion constant of (425 ± 0.01) µm^2^/s in water at 25 °C [36]. Diffusion coefficients D can be related to temperature T, viscosity η(T) and hydrodynamic radius r by the Stokes-Einstein equation D(T) = k_B_T/(6πη(T)r). Viscosity was estimated by η(T) = a∙exp(−b/(T−c)) with a = 2.414∙10^−5^ Pas, b = 247.8 K, and c = 140 K (Picoquant application note). Calibration and SMALP measurements at different temperatures were corrected by these equations. The final hydrodynamic radius was estimated by the measured diffusion coefficient and the Stokes–Einstein equation.

### 2.10. Transmission Electron Microscopy

Copper grids (300 mesh formvar/carbon-coated) (Ted Pella, Redding, CA, USA) were hydrophilized by glow discharge (−20 mA, 45 s) with a Pelco EasyGlow apparatus (Ted Pella, USA). Fresh protein samples (3 µL) were placed onto the grid and incubated at room temperature (RT) for 30 s. Excess of the sample was removed with filter paper. Grids were stained twice, using 1% aquatic uranyl acetate solution for 30 s at RT, and air-dried.

Micrographs were acquired using an analytical transmission electron microscope Jem-2100 (Jeol, Akishima City, Japan) equipped with a 2K × 2K CCD camera Ultrascan 1000XP (Gatan, Pleasanton, CA, USA). The microscope operated at 200 kV in a low dose mode, with a magnification ×40,000 (2.5 Å/pix) and defocus 0.5–1.9 µm. Images were acquired automatically with SerialEM3.8.0 software [37].

### 2.11. Image Processing

To obtain the 2D projections of purified Wsc1 protein molecules, 107712 particles were selected automatically from the 870 EM images using crYOLO neural network [38]. Particles were windowed into 80 × 80 pixel images and analyzed in the EMAN2.3 suite [39,40]. The 3D reconstruction of Wsc1 was accomplished with RELION 2.0 [41]. Briefly, 2D class averages were produced by classification images of Wsc1 particles. For each complex, 50 classes were produced, and 25 iterations were used to increase the signal-to-noise ratio. After classification, all classes were ranked according to quality. The worst 2D classes (representing approximately 5% of all particles) were discarded. For the 3D reconstruction only those particles were selected, that represent elongated Wisc1-GFP structure. In total, 46,500 particles were subjected to 3D classification in RELION 2.0. It produced three 3D classes with the largest number of particles and highest signal-to-noise, that were chosen to build 3D models. C1 symmetry was used for all reconstructions, including the final one from the class 1 (16818 images) obtained at a resolution of 18 Å (Figure S2). The atomic structures of the GFP (Protein Data Bank ID: 4ogs) and the N-terminal homologue-KRM1_WSC_ domain (Protein Data Bank ID: 5fws) were fit into the density map using UCSF Chimera [42].

### 2.12. Bioinformatics Analysis

For modelling the 3D structure of WSC1, we searched for its structural homologs by performing searches with the Position-Specific Iterative Basic Local Alignment Search Tool (PSI-BLAST) (https://blast.ncbi.nlm.nih.gov/Blast.cgi [43]) against all the structures in Protein Data Bank PDB (https://www.rcsb.org/ [44]). The validity of the search results was checked by comparison with the PROSITE database (https://prosite.expasy.org/ [45]).

## 3. Results

### 3.1. Construction of a Yeast Strain for Stable Expression of a Tagged WSC1 Construct

**Figure 1:**
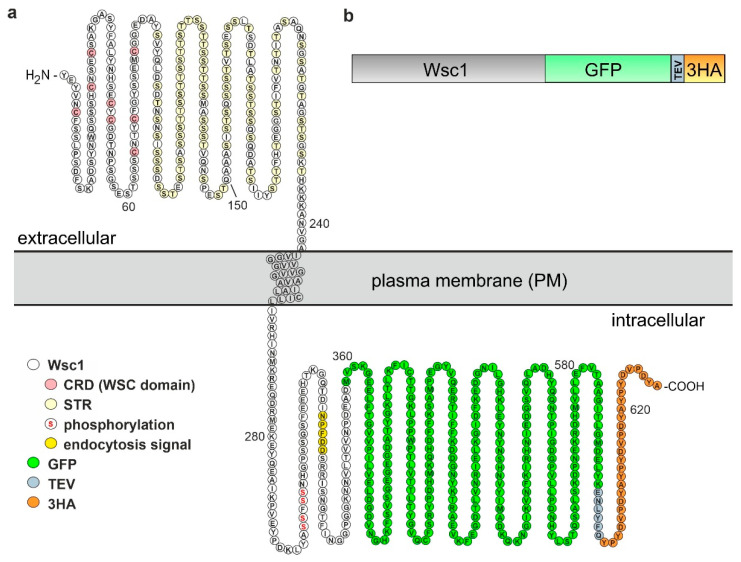
Schematic representations of the Wsc1 fusion construct employed in this work. (**a**) Primary structure of the sensor fusion protein indicating its orientation within the yeast plasma membrane, designed with Protter according to [47] and modified manually. Colour codes within the Wsc1 sequence highlight the eight cysteine residues characteristic of the WSC- or cysteine-rich domain (CRD), the residues believed to be glycosylated in the serine/threonine-rich region (STR; [6]), the serine residues within the cytoplasmic tail which could be phosphorylated [48], and the signal motif for endocytosis [46]. (**b**) Simplified overview of different protein sequences fused to the C-terminal end of Wsc1.

In previous works we employed a yeast strain carrying a *WSC1* gene tagged at its native locus with the coding sequence for the green fluorescent protein (GFP) to produce a C-terminally tagged, fully functional sensor fusion protein [46]. While preliminary experiments demonstrated that this construct was suitable to detect the detergent-free incorporation of the sensor from the yeast plasma membrane into a styrene maleic acid (SMA) copolymer, it raised the problem that the preparation of SMA lipoproteins (SMALPs) contained a mixture of membrane proteins, which only in a small fraction represented the Wsc1 sensor. In order to provide a means to enrich the latter fraction, we decided to additionally tag the sensor GFP-fusion at the C-terminal end with the recognition sequence for the TEV protease followed by three copies of the hemagglutin antigen (Figure 1; [10]; note that the TEV sequence was inserted to further facilitate purification, which turned out not be necessary in the course of this work). Spheroplasts prepared from this yeast donor strain were then used as a source of membrane proteins to obtain nanosized SMALPs to be characterized by a set of biophysical approaches, as outlined below.

### 3.2. Enrichment of Wsc1-GFP in SMALPs

**Figure 2:**
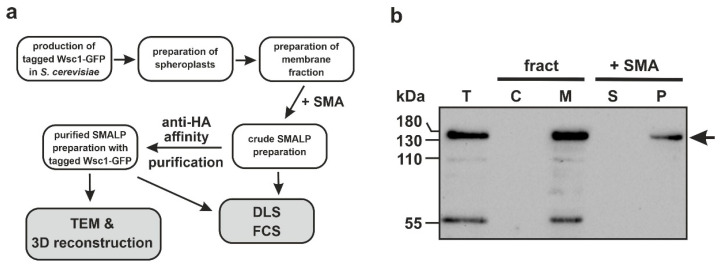
Purification and examination of styrene maleic acid lipoproteins (SMALPs) containing Wsc1-green fluorescent protein (GFP). (**a**) General scheme of the experimental strategy followed in this work. (**b**) Immunoblot analysis of cell fractionation and solubilization of the sensor fusion in the styrene maleic acid (SMA) polymer. T = total cell lysate; fract = cell fractionation with C = soluble cytosolic proteins, M = membrane-bound, pelleted fraction; +SMA = solubilization with SMA with S = supernatant (i.e., SMALPs), P = pellet with debris. The arrow indicates the signal of the presumably highly glycosylated sensor, according to [50].

In order to purify the tagged Wsc1 protein for further characterization, the general scheme depicted in Figure 2a was followed. Thus, we first prepared yeast spheroplasts from logarithmically growing cells by digesting the cell wall with the Zymolyase glucanase preparation. This gentle treatment prevents the liberation of endogenous intracellular proteases caused by mechanical damage, which should also help to protect the Wsc1 membrane sensor from proteolytic degradation [49]. After disruption of the spheroplasts with ultrasound, the membrane fraction was harvested by centrifugation and resuspended in Tris-HCl buffer with sodium chloride. For extraction of the tagged sensor, the membrane suspension was then incubated with a 2.5% SMA copolymer solution and subsequently clarified by centrifugation. Finally, the supernatant containing SMALP-preparation was loaded onto an anti-HA tag affinity column and eluted to obtain Wsc1-GFP purified in SMALPs.

Western blotting and immunological detection (Figure 2b) verified the presence of the Wsc1-GFP protein in the whole cell membranes, after cell fractionation and membrane solubilization. Wsc1-GFP was exclusively retained in the membrane fraction (Figure 2b, lane M), as demonstrated by comparison to the total cell lysate (Figure 2b, lane T). The protein appeared in a major band at approximately 130 kDa on the immunoblot probed with anti-HA (Figure 2b), which presumably represents the highly glycosylated sensor [50]. A minor signal at 55 kDa probably reflects a proteolytic cleavage product, which was eliminated by the copolymer treatment (note that the Wsc1 fusion protein has a calculated molecular mass of 73 kDa). The smaller band is unlikely to represent a GFP dimer, as this would dissociate in the denaturing gel conditions and is not likely to form in the first place with the GFP employed in our construct [51]. With regard to the glycosylated sensor, a significant portion remained in the original membrane fraction (Figure 2b, lane P) after treatment with the SMA copolymer, while the amount of Wsc1-GFP solubilized was below the sensitivity threshold of the immunoblot (Figure 2b, lane S), it could be detected by fluorescence spectroscopy (as explained below).

### 3.3. Biophysical Characterization of Crude SMALP Preparations

**Figure 3:**
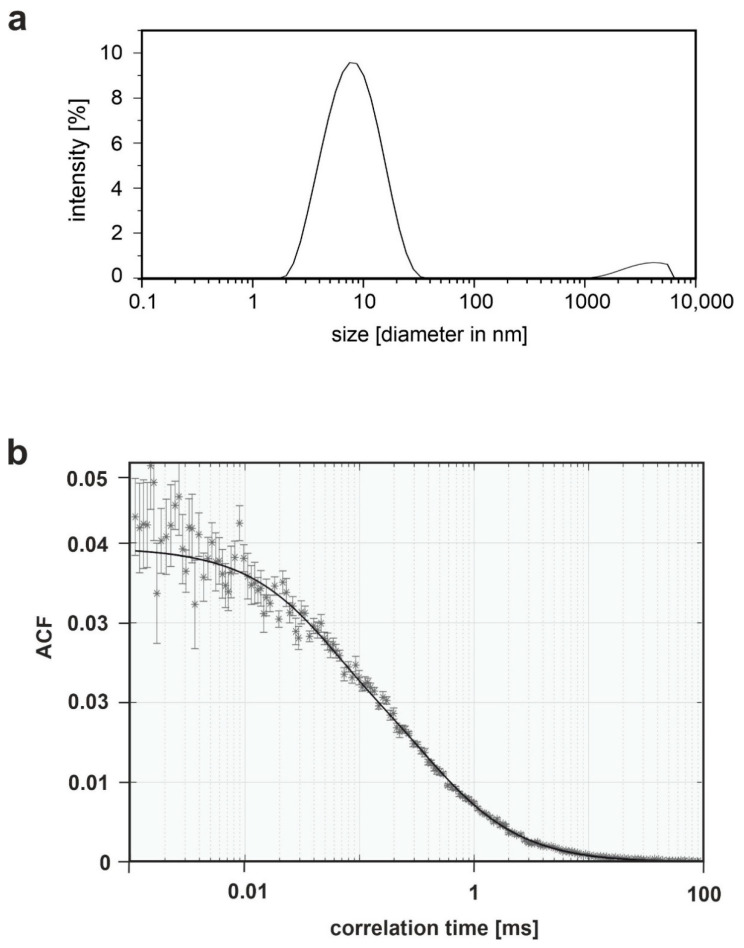
Quality assessment of crude SMALP preparations. (**a**) Representative image of size distribution of a SMALP preparation after membrane solubilization based on dynamic light scattering (DLS) data. (**b**) Representative example of a Fluorescence Correlation Spectroscopy (FCS) analysis. The curve was fitted by a model considering triplet-state blinking (black line). Calculations given in the text were based on at least three such curves obtained for four different SMALP preparations, each, which displayed similar kinetics. ACF = autocorrelation function.

Dynamic light scattering (DLS) was employed to analyze the size distribution of the SMA copolymer particles in the supernatant fraction obtained after membrane solubilization (further referred to as soluble membrane fraction). These data confirmed the presence of monodisperse nano-sized particles (Figure 3a). Supporting data from previous works, their intensity-weighted diameter was estimated to be in the 10-nm range [14,15,22,52]. More importantly, we thus demonstrated that incubation of the yeast cellular membrane fraction with the SMA copolymer in a ratio of 3:1 indeed yielded nano-sized SMALPs. 

These SMALPs were then subjected to fluorescence correlation spectroscopy (FCS), which allows the robust determination of diffusion constants at nanomolar concentrations following fluorescent labels, in order to determine their content of Wsc1-GFP. First, the identity of the GFP signals was verified by determination of the fluorescence emission spectrum. From the diffusion constants obtained in the FCS, hydrodynamic radii were estimated using the Stokes-Einstein equation, assuming a spherical shape of the particles. Samples were then subjected to robust FCS measurements based on their GFP fluorescence, using appropriate dilutions to nanomolar concentrations if necessary. Typical FCS autocorrelation results are shown in Figure 3b. The fit indicates a monodisperse population of SMALP particles undergoing three-dimensional free diffusion. Furthermore, fitting the experimental autocorrelation function revealed an average particle diffusion coefficient of 53.0 ± 3.0 µm^2^/s (± standard error). The average hydrodynamic particle radius, r, was 4.9 ± 0.3 nm and the Wsc1-GFP concentration was approximately 30 nM. We took these data as strong evidence that the Wsc1-GFP protein was indeed present in the SMALP preparation after membrane solubilization, basically carrying a single sensor molecule per particle. The latter assumption was confirmed by the TEM pictures taken of the purified SMALPs as described below. Moreover, the FCS values obtained for the size of the nanoparticles correlated well with the DLS data discussed above.

### 3.4. Biophysical Characterization of Affinity-Purified SMALPs with Wsc1-GFP

We assumed that the soluble membrane fraction, i.e., the supernatant fraction obtained after membrane solubilization by incubation with the SMA copolymer, contained both SMALPs loaded with Wsc1-GFP and sensor-free SMALPs carrying other yeast membrane proteins. The fraction containing the GFP-tagged sensor was further purified by application of the supernatant to an anti-HA affinity column. Similar to the soluble membrane fraction preparation shown in Figure 2b, Wsc1-GFP concentrations in the purified preparations remained below the detection limit in immunoblotting and were also too low for reliable DLS measurements (data not shown). However, a GFP signal could be detected and characterized in the purified SMALPs by FCS. The diffusion coefficient was close to the one determined above for the non-purified SMALPs, with 50.0 ± 4.6 µm^2^/s as compared to 53.0 ± 3.0 µm^2^/s, respectively. Likewise, the corresponding hydrodynamic radius, r, of 4.9 ± 0.3 nm remained unaltered. This confirmed that affinity purification did not affect the physicochemical properties of the SMALP-embedded sensor.

### 3.5. Three-Dimensional Modelling of the Tagged Wsc1 Sensor Based on Negative-Stain Transmission Electron Microscopy and Bioinformatic Analyses

**Figure 4:**
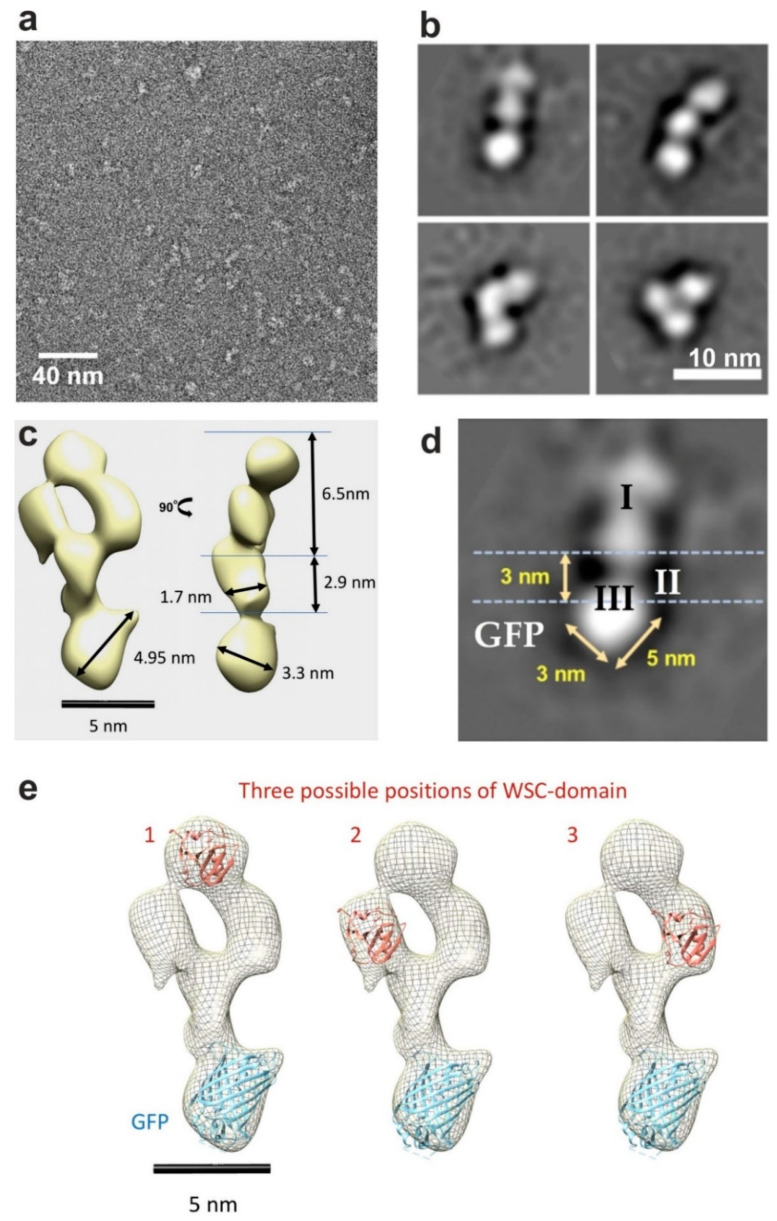
Construction of a three-dimensional model of the tagged Wsc1 sensor. (**a**) Images of the purified Wsc1-carrying SMALP preparation obtained by negative-stain transmission electron microscopy. (**b**) Four representative examples of two-dimensional class images of the Wsc1-GFP sensor fusion. 2D classes that are representative of those used for the 3D reconstruction are shown in the two images of the upper row. (**c**) Three-dimensional reconstruction of the sensor structure. Measurements were performed in ImageJ [53]. The two views depicted in were obtained by successive 90° rotations around the vertical axis. (**d**) 2D class-average of the extended Wsc1-GFP construct with designated domains, depicting their dimensions and the proposed position of the membrane. I, II, and III designate the proposed extracellular, transmembrane, and cytosolic domains, respectively. (**e**) Possible locations of homologous domains with a resolved crystal structure. The structure of GFP (pdb ID 4OGS) was fitted into the presumed cytosolic extension of the protein (blue ribbon) and the crystal structure homologous to the WSC domain of KRM1 (pdb ID 5FWS) would fit into any of the three cytosolic densities shown (salmon ribbon).

Finally, affinity-purified SMALPs loaded with Wsc1-GFP were analyzed by negative-stain transmission electron microscopy (nsTEM). As shown in the exemplary image in Figure 4a, the particles observed were mostly single, rather homogeneous, and did not form larger aggregates. A total of 107,000 single particle images were then acquired and first classified for their appearance in two dimensions (2D). Some of the class images displayed elongated structures with a length of approximately 10–15 nm, with three clearly distinguishable segments (Figure 4b). Nevertheless, the particle geometry was highly dynamic, with some showing an extended conformation (top row in Figure 4b), while others adopted a ‘folded’ shape (bottom row in Figure 4b).

A PSI-BLAST search immediately revealed sequence similarity between residues 28–80 of the mature Wsc1 protein and a sequence stretch of the human KREMEN1 receptor. All eight functionally important cysteine residues matched (see alignments from run 1 presented in supplementary materials File S1, with the first four KREMEN1 receptor sequences). This region corresponds to the WSC domain in the KREMEN1 receptor structure (PDB ID 5FWS [54]) named after the homologous region near the N-terminus of Wsc1 (PROSITE entry PS51212; WSC). This first analysis revealed no further obvious candidates that could provide structural information for other parts of Wsc1.

However, the second iteration of the PSI-BLAST search (supplementary File S2) detected a sequence similarity between the following 200 residues of Wsc1 with the Ebola virus membrane-bound glycoprotein (GP), for which the structure of its soluble variant has been resolved [55]. Although the overall sequence similarity is fairly low, both proteins are highly enriched in serine and threonine residues in this region, which are highly glycosylated and typically inherent to intrinsically disordered proteins [56].

Indeed, the structure of Ebola virus GP (PDB ID 5KEL) displays short, disordered α-helices and β-strands [55]. It should be noted that the folding of these regions depends on the interaction of the respective proteins with physiological partners and can be determined only in their presence (see [57,58] for recent reviews). Thus, the Ebola virus GP structure was resolved only in a complex with specific antibodies [55], which impeded its use in the modelling of Wsc1. In any case, the periplasmic part of Wsc1 is built of two structural segments: the N-terminal domain stabilized by disulfide bridges forming the known WSC domain (further described in the next paragraph), and the following segment, which probably has a variable, disordered structure sensitive to the degree of its glycosylation [56]. This could cause the second segment to look somewhat fuzzier on processed EM images.

Since the position of the membrane-crossing α-helix could only be identified in images with the extended conformation (Figure 4c,d), only those were considered for extraction by RELION 2.0 and employed to calculate the three-dimensional (3D) structure of the protein (Figure 4c). The remaining 46,500 single particles which met this criterion then served to generate the 3D reconstructions of Wsc1-GFP in an extended conformation with C1 symmetry, as depicted in Figure 4c.The resulting model reflected the three putative functional parts of the Wsc1 sensor (Figure 4d). We used the 3D structure of the WSC-homologous domain of KREMEN1 (residues Gly129-Thr220; PDB ID 5FWS [54]) for modelling the shape of the N-terminal Wsc1 part. The rest of the extracellular part was assumed to be entropically unstable and to lack a defined structure, while its exact architecture is unknown (note that the overall size of this domain is compatible with any of three reconstructed extracellular periplasmic densities). The WSC-like domain of KRM1 may fit in three different ways (Figure 4e). The entire extracellular part then has a clasp-like appearance and is connected to the transmembrane domain (TMD) via a hinge, which confers flexibility relative to the TMD. With approximately 1.7 nm × 2.9 nm the TMD appears to be slightly broader than expected from the primary sequence of a single alpha-helix (Figure 4c,d). This could be attributed to the varying position of GFP and the extracellular part relative to the transmembrane helix within different lipodiscs and/or to a distortion from lipodisc surrounding the membrane domain.

The size of the third, bulkier domain fits the short cytosolic sequence of Wsc1 fused to the GFP ß-barrel. The crystal structure of GFP [59] was thus modelled into this domain, leaving space for the cytosolic tail of the sensor. As seen in Figure 4e, the GFP does not fit perfectly, leaving an additional thumb-like projection on the right-hand side close to the inner membrane surface, which may correspond to the folded cytosolic tail of Wsc1 (60 amino acids in length).

## 4. Discussion

Recently, the amphipathic styrene-maleic acid (SMA) and SMA-related copolymers became a promising tool for membrane protein purification. Here, we have reported the application of SMA in the solubilization of the membrane spanning sensor protein Wsc1 as an important constituent of the native plasma membrane from the model yeast *S. cerevisiae*. Wsc1 is a major player in the activation of the cell wall integrity (CWI) pathway, which regulates cell wall remodeling during periods of extracellular stress, mating and normal cell growth [5]. We took advantage of the GFP-moiety of a tagged functional protein employed in previous works [10,46] and equipped it with an additional 3xHA-tag for affinity purification. By combining the ease of genetic engineering in yeast cells for specific experimental purposes with the use of advanced biophysical methods, we were able to successfully isolate and purify the tagged sensor in a detergent-free manner and in a semi-native lipid environment. This allowed a compilation of images obtained by negative-stain electron microscopy (nsTEM), from which a first three-dimensional structural model of Wsc1 was deduced.

**Figure 5:**
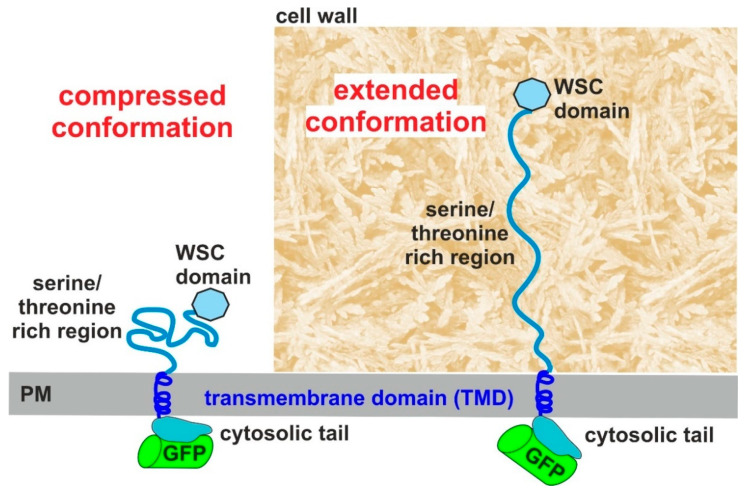
Proposed conformations of the Wsc1 sensor and position of its cysteine-rich domain (WSC domain) in the SMALP preparations (left) and in living yeast cells (right). PM = plasma membrane.

The model is consistent with a tripartite organization of Wsc1, previously suggested based on its primary sequence and mechanical features revealed by single-molecule atomic force microscopy (AFM, [7]). These three parts correspond to the relatively large extracellular region, a single transmembrane domain, and a relatively short cytoplasmic tail at the C-terminus. Due to its high flexibility in the nsTEM images, modelling of the extracellular region is most problematic and probably does not closely reflect the in vivo conformation. The models deduced from our 3D class images (Figure 4c and Figure S1) exhibit a relatively large extracellular domain (approximately 6.5 × 6.4 nm) forming a horseshoe, attached to the smaller TMD density of 1.7 × 2.9 nm. The N-terminal cysteine-rich WSC-domain seems to be the only folded part of the whole extracellular domain of Wsc1, which is expected to form disulfide bridges and interact with the carbohydrate chains of the cell wall glucans (Figure 5; [9,10]). It is tempting to speculate that the polysaccharide chains could go through the hairpin structure and thus pull the sensor if they are distorted. Consistent with the proposed function as a mechanosensor [6], the force could be generated by dislocation of the polysaccharide chains, as a consequence of stretching caused by changes in turgor in reaction to medium osmolarity or by cell wall damage. The hairpin then could be easily imagined to break open under extensive mechanical stress thus allowing the sensor to be internalized by the rapid endocytosis observed in live yeast cells [46,60] solving the puzzle of how mechanical interaction with the cell wall is compatible with sensor internalization. Clearly, evidence for such a mechanism would require visualization of the protein associated with the cell wall, for which the methodology does not yet exist. The serine/threonine-rich region (STR), that is likely to be intrinsically disordered [56], has been proposed to penetrate the yeast cell wall in the form of a nanospring, which could stretch for 42 nm as shown by AFM measurements [7]. In fact, the disordered arrangement of the serine/threonine rich segment, which is held together by entropic forces, would be consistent with a spring-like behavior. Consistent with this view, our estimates based on this model now would predict that this 200 aa-long segment would cover some 50 nm distance, if completely stretched (Figure 5).

We assume that the shrinking and reduction of the external length to about 6–7 nm, as observed for the EM images, is a manifestation of an entropically collapsed structure of the nanospring, which, otherwise, could be considerably stretched by interaction of the N-terminal WSC-domain with the cell wall in living yeast cells under standard conditions, and even more though upon perturbances of the cell surface (Figure 5). As stated above, the compressed conformation(s) of the periplasmic domain may be a consequence of the extraction of full-length Wsc1 from the plasma membrane and foremost of preventing its interaction with the yeast cell wall, due to the Zymolyase treatment and transfer to SMA. The ability for stretching in live yeast cells greatly enhances the sensitivity of the sensor towards perturbations in either the cell wall or the plasma membrane, which would be transmitted through the transmembrane part to the cytosolic tail to trigger CWI signaling, supporting the role of Wsc1 as a mechanosensor [7]. It should be noted that the external domain also confers the species-specific properties of CWI signaling in different yeasts, as deduced from heterologous expression studies of the related Mid2 sensor [61]. It would thus be interesting to resolve the structures of Wsc1 homologues from other yeasts and/or those of Mid2, which lack a WSC domain, in future studies.

The cytosolic tail of Wsc1 is of utmost importance in signal transduction to the CWI pathway. It has been shown to interact with the guanine nucleotide exchange factor (GEF) Rom2 triggering the downstream signaling cascade [48]. Interesting, a tyrosine residue (Y282 in Figure 1a) was shown in the latter work to mediate this interaction, which is counteracted by phosphorylation of downstream serine residues, which could lead to an electrostatic repulsion from the positively charged inner leaf of the PM. The cytosolic domain also mediates the highly dynamic intracellular distribution of Wsc1, which is constantly subjected to endocytosis, depending on an NPFXD motif [46,60,62]. Endocytosis of the sensor also depends on its lipid environment within the PM, as depletion of phosphoinositol-4,5-bisphosphate results in a massive accumulation of Wsc1 and other CWI signaling components in recycling endosomes [63]. Moreover, early studies indicated that the Wsc-type sensors reside in so-called lipid rafts, i.e., membrane compartments defined by their resistance towards solubilization by Triton X100 [50]. However, apart from these two reports indicating their functional influence, the exact lipid composition of the membrane locally surrounding Wsc1 sensors is not known.

In our 3D reconstruction, the cytosolic part probably best reflects the natural conformation in living yeast cells, as it is in the natural aqueous environment and relatively short. As evident from Figure 4 and Figure 5, the thumb-like structure of the cytosolic tail seems to align with the inner leaf of the membrane. Its location is consistent with previous observations, that Wsc1 function is not affected by fusion to the GFP tag [10,60]. Moreover, as indicated at the left hand side of Figure 5, it could be dislocated from this position by external stress and/or by electrostatic repulsion due to the observed phosphorylation of serine residues [48]. This then would enable the sensor to interact with the downstream components of the CWI pathway or other modification factors. The model also explains how slight modifications in the angle of the hinge region connecting the TMD to the intracellular part, e.g., by pulling it towards the lipid bilayer, could result in dramatic changes in the exposure of the cytosolic tail. Obviously, such conformational changes would be brought about by the interaction of the extracellular part of Wsc1 with the yeast cell wall, thus transmitting perturbations through the TMD to the interior of the cell.

This work provides interesting insights into the structure of the Wsc1 sensor and confirms the importance of studying TM receptors and sensors in lipid-based, native-like environments. For instance, the monodisperse purified SMALPs containing the sensor had an average diameter of only 10 nm, accommodating only single sensor molecules. However, Wsc1 has been shown to cluster in large complexes within the plasma membrane of living yeast cells and was suggested to form at least dimers, both homodimers and heterodimers with other Wsc-type sensors, as deduced from bimolecular fluorescence interactions [8]. An extraction method for sensor multimers would thus be the next challenge.

Diisobutylene/maleic acid (DIBMA) copolymers, which differ from SMA copolymers in the non-aromatic nature of their hydrophobic groups, can also solubilize membrane proteins directly from a lipid bilayer. However, the size of DIBMA/lipid particles (DIBMALPs) can be varied in a broader range than that of SMALPs, thus reaching dimensions of up to 50 nm [64,65,66]. In future studies it would be interesting to employ DIBMA copolymers for the extraction of dimers and larger sensor complexes from the yeast plasma membrane into large sized polymer/lipid particles. Furthermore, the interaction between yeast cell wall extracts and SMALP-embedded sensors should be studied, to determine whether Wsc1 can access its extended conformation in the presence of cell wall polysaccharides. 

Nevertheless, this work demonstrated the applicability of a detergent-free extraction and purification procedure, which is suitable not only for other membrane proteins of *S. cerevisiae*, but also of pathogenic yeasts and other fungi. Choosing essential members of such membrane proteins to study their 3D structure could thus provide a new angle for the design of antifungal agents, urgently needed in medicine and agriculture.

## Data Availability

Original datasets, strains and constructs employed in this work are freely available upon request to the academic community for research purposes, only.

## Supplementary Materials

The following materials are available online at https://www.mdpi.com/2309-608X/7/2/118/s1, File S1: Report on the initial PSI-BLAST search with the Wsc1 sequence as query. File S2: Report on the second iteration PSI-BLAST search with the Wsc1 sequence as query. The Ebola virus GP alignment is marked yellow. Figure S1: Additional 3D classes of Wsc1-GFP construct, calculated in RELION2.0 [43]. For each reconstruction two orientations are shown, that differ by rotation for 90 degrees around vertical axis. Bar–5 nm; Figure S2: Fourier shell correlation plot for the 3D reconstruction in Figure 4c.
