# Supplementary material for: A Three-Dimensional Model of the Yeast Transmembrane Sensor Wsc1 Obtained by SMA-Based Detergent-Free Purification and Transmission Electron Microscopy
Source: J Fungi (Basel). 2021 Feb 5;7(2):118. doi: 10.3390/jof7020118 (PMC7915640; doi:10.3390/jof7020118)
Supplement: Supplementary file 1 [file jof-07-00118-s001.pdf]

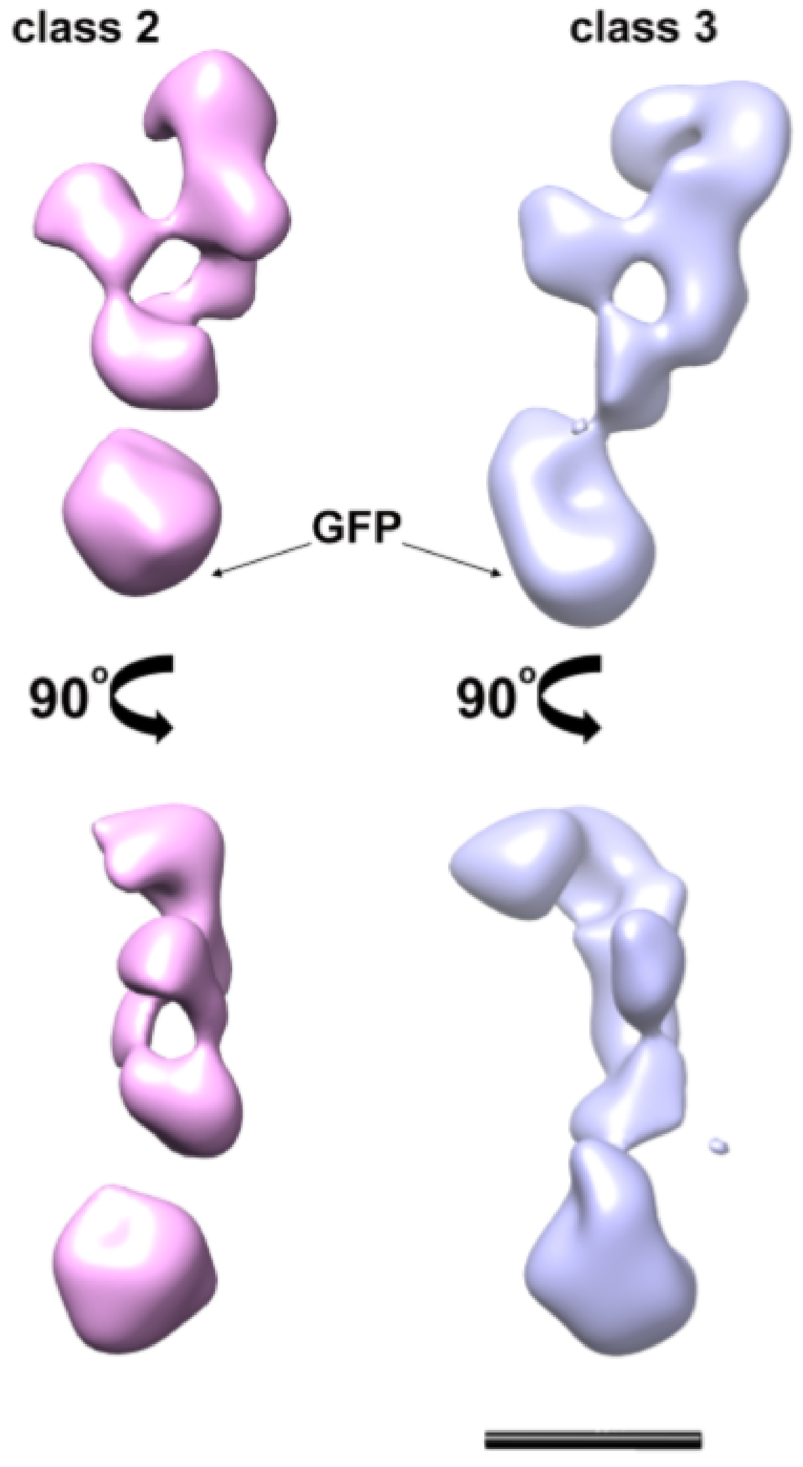

Figure S1: Additional 3D classes of Wsc1-GFP, calculated with RELION2.0 [43]. Two orientations are shown for each reconstruction, which differ by rotation for 90 degrees around the vertical axis. The size bar corresponds to 5 µm.

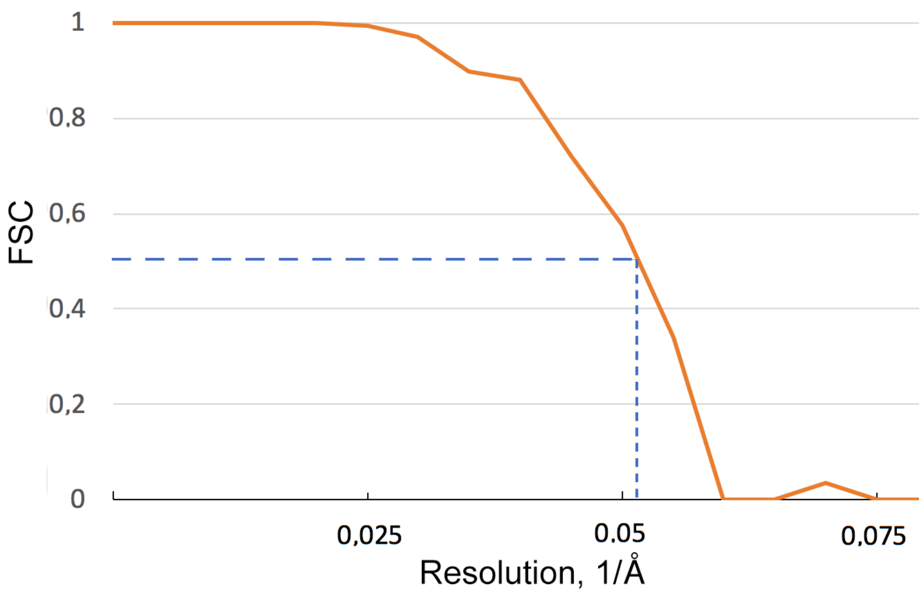

Figure S2. Fourier shell correlation plot for the 3D reconstruction in Fig. 4C.

COVID-19 is an emerging, rapidly evolving situation.
Get the latest public health information from CDC: https://www.coronavirus.gov
Get the latest research information from NIH: https://www.nih.gov/coronavirus
Find NCBI SARS-CoV-2 literature, sequence, and clinical content: https://www.ncbi.nlm.nih.gov/sars-cov-2/

BLAST ® » **blastp suite** » **results for RID-ZHDZDEH6013**

**Job Title**	gb|AJT71945.1| ...
**RID**	ZHDZDEH6013 Search expires on 01-10 02:52 am
**Program**	PSI-BLAST Iteration 1
**Database**	pdb
**Query ID**	AJT71945.1
**Description**	Slg1p [Saccharomyces cerevisiae YJM195] ...
**Molecule type**	amino acid
**Query Length**	378

Run PSI-Blast iteration 2

Number of sequences Run

Descriptions

Sequences with E-value BETTER than threshold

Description	Scientific Name	Max Score	Total Score	Query Cover	E value	Per. Ident	Acc. Len	Accession	Select for PSI blast	Used to build PSSM	Newly added

Run PSI-BLAST Iteration 2 with max number of sequences Run

Sequences with E-value WORSE than threshold

Description	Scientific Name	Max Score	Total Score	Query Cover	E value	Per. Ident	Acc. Len	Accession	Select for PSI blast	Used to build PSSM	Newly added
Structure of Coxsackievirus A10 complexed with its receptor KREMEN1 [Homo sapiens]	Homo sapiens	36.5	36.5	14%	0.047	32.14%	378	6SNW_E (scored below threshold on previous iteration)	Select seq 6SNW_E for PSI blast		
Cryo-EM structure of mature Coxsackievirus A10 in complex with KRM1 at pH 7.4 [Homo sapiens]	Homo sapiens	36.5	36.5	14%	0.048	32.14%	375	7BZT_E (scored below threshold on previous iteration)	Select seq 7BZT_E for PSI blast		
Wnt modulator Kremen crystal form I at 1.90A [Homo sapiens]	Homo sapiens	36.2	36.2	14%	0.053	32.14%	406	5FWS_A (scored below threshold on previous iteration)	Select seq 5FWS_A for PSI blast		
Wnt modulator Kremen in complex with DKK1 (CRD2) and LRP6 (PE3PE4) [Homo sapiens]	Homo sapiens	34.8	34.8	14%	0.14	32.14%	293	5FWW_B (scored below threshold on previous iteration)	Select seq 5FWW_B for PSI blast		
Solution NMR Structure of the serine-rich domain of hEF1 (Enhancer of filamentation 1) from Homo sapiens, Northeast Structural Genomics Consortium Target HR5554A [Homo sapiens]	Homo sapiens	32.1	32.1	8%	0.56	46.88%	176	2L81_A (scored below threshold on previous iteration)	Select seq 2L81_A for PSI blast		
Structure of human voltage-gated sodium channel Nav1.7 in complex with auxiliary beta subunits, huwentoxin-IV and saxitoxin (Y1755 up) [Homo sapiens]	Homo sapiens	31.6	31.6	10%	1.2	32.50%	215	6J8G_C (scored below threshold on previous iteration)	Select seq 6J8G_C for PSI blast		
Solution structure of integrin b2 monomer tranmembrane domain in bicelle [Homo sapiens]	Homo sapiens	28.6	28.6	10%	1.5	40.00%	52	5ZAZ_A (scored below threshold on previous iteration)	Select seq 5ZAZ_A for PSI blast		
Structure of Tra1 subunit within the chromatin modifying complex SAGA [Komagataella pastoris]	Komagataella pastoris	32.1	32.1	29%	1.8	22.12%	3825	5OEJ_B (scored below threshold on previous iteration)	Select seq 5OEJ_B for PSI blast		
The structure of an Allene Oxide Synthase reveals a novel use for a catalase fold [Plexaura homomalla]	Plexaura homomalla	30.4	30.4	7%	4.5	43.75%	374	1U5U_A (scored below threshold on previous iteration)	Select seq 1U5U_A for PSI blast		
RNA Polymerase II from Komagataella Pastoris (Type-1 crystal) [Komagataella phaffii GS115]	Komagataella phaffii GS115	30.4	30.4	28%	5.4	26.61%	1743	5X4Z_A (scored below threshold on previous iteration)	Select seq 5X4Z_A for PSI blast		
Allene oxide synthase 8R-lipoxygenase from Plexaura homomalla [Plexaura homomalla]	Plexaura homomalla	30.4	30.4	7%	5.4	43.75%	1066	3DY5_A (scored below threshold on previous iteration)	Select seq 3DY5_A for PSI blast		
Solution NMR Structure of APP TMD [Homo sapiens]	Homo sapiens	26.3	26.3	7%	6.8	41.38%	30	6YHF_A (scored below threshold on previous iteration)	Select seq 6YHF_A for PSI blast		
Allophanate Hydrolase Complex from Mycobacterium smegmatis, Msmeg0435-Msmeg0436 [Mycolicibacterium smegmatis MC2 155]	Mycolicibacterium smegmatis MC2 155	29.5	29.5	7%	7.3	40.74%	318	3MML_A (scored below threshold on previous iteration)	Select seq 3MML_A for PSI blast		
Solution NMR Structure of APP V44M mutant TMD [Homo sapiens]	Homo sapiens	26.0	26.0	7%	8.4	41.38%	30	6YHP_A (scored below threshold on previous iteration)	Select seq 6YHP_A for PSI blast		
Structure of amyloid precursor protein's transmembrane domain [Homo sapiens]	Homo sapiens	26.3	26.3	7%	8.5	41.38%	43	2LLM_A (scored below threshold on previous iteration)	Select seq 2LLM_A for PSI blast		

Graphic Summary

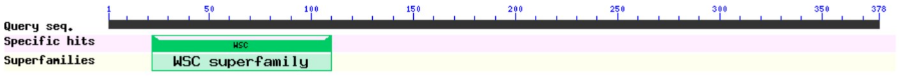

Distribution of the top 15 Blast Hits on 15 subject sequences

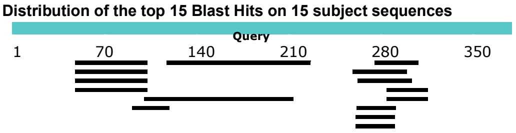

Alignments

Alignment view Pairwise ☐ CDS feature Restore defaults

Structure of Coxsackievirus A10 complexed with its receptor KREMEN1 [Homo sapiens]
Sequence ID: **6SNW_E** Length: 378 Number of Matches: 1
Range 1: 130 to 184

Score	Expect	Method	Identities	Positives	Gaps	Frame
36.5 bits(109)	0.047()	Compositional matrix adjust.	18/56(32%)	31/56(55%)	4/56(7%)	

```
Query  49    CNSECSAKGASYFALYNHSECYCGDTNP---SGSESTSSSCNTYCFGYSSEMCGGE  101
             C S C ++   +  + +   C+CG+ NP      E+ S+ CN+ CFG  ++ CGG+
Sbjct  130   CISFCRSQRFKFAGMESGYACFCGN-NPDYWKYGEAASTECNSVCFGDHTQPCGGD  184
```

Cryo-EM structure of mature Coxsackievirus A10 in complex with KRM1 at pH 7.4 [Homo sapiens]
Sequence ID: **7BZT_E** Length: 375 Number of Matches: 1
Range 1: 127 to 181

Score	Expect	Method	Identities	Positives	Gaps	Frame
36.5 bits(109)	0.048()	Compositional matrix adjust.	18/56(32%)	31/56(55%)	4/56(7%)	

```
Query  49    CNSECSAKGASYFALYNHSECYCGDTNP---SGSESTSSSCNTYCFGYSSEMCGGE  101
             C S C ++   +  + +   C+CG+ NP      E+ S+ CN+ CFG  ++ CGG+
Sbjct  127   CISFCRSQRFKFAGMESGYACFCGN-NPDYWKYGEAASTECNSVCFGDHTQPCGGD  181
```

Wnt modulator Kremen crystal form I at 1.90A [Homo sapiens]
Sequence ID: **5FWS_A** Length: 406 Number of Matches: 1
Range 1: 158 to 212

Score	Expect	Method	Identities	Positives	Gaps	Frame
36.2 bits(108)	0.053()	Compositional matrix adjust.	18/56(32%)	31/56(55%)	4/56(7%)	

```
Query  49    CNSECSAKGASYFALYNHSECYCGDTNP---SGSESTSSSCNTYCFGYSSEMCGGE  101
             C S C ++   +  + +   C+CG+ NP      E+ S+ CN+ CFG  ++ CGG+
Sbjct  158   CISFCRSQRFKFAGMESGYACFCGN-NPDYWKYGEAASTECNSVCFGDHTQPCGGD  212
```

Wnt modulator Kremen in complex with DKK1 (CRD2) and LRP6 (PE3PE4) [Homo sapiens]
Sequence ID: **5FWW_B** Length: 293 Number of Matches: 1
Range 1: 118 to 172

Score	Expect	Method	Identities	Positives	Gaps	Frame
34.8 bits(103)	0.14()	Compositional matrix adjust.	18/56(32%)	31/56(55%)	4/56(7%)	

```
Query  49    CNSECSAKGASYFALYNHSECYCGDTNP---SGSESTSSSCNTYCFGYSSEMCGGE  101
             C S C ++   +  + +   C+CG+ NP      E+ S+ CN+ CFG  ++ CGG+
Sbjct  118   CISFCRSQRFKFAGMESGYACFCGN-NPDYWKYGEAASTECNSVCFGDHTQPCGGD  172
```

Solution NMR Structure of the serine-rich domain of hEF1 (Enhancer of filamentation 1) from Homo sapiens, Northeast Structural Genomics Consortium Target HR5554A [Homo sapiens]
Sequence ID: **2L81_A** Length: 176 Number of Matches: 1
Range 1: 79 to 110

Score	Expect	Method	Identities	Positives	Gaps	Frame
32.1 bits(94)	0.56()	Compositional matrix adjust.	15/32(47%)	20/32(62%)	0/32(0%)	

```
Query  273   VVGAVAIALCILLIVRHINMKREQDRMEKEYQ  304
             V GAVA A C+  ++ H  MKRE  R+E  +Q
Sbjct  79    VKGAVANAACLPELILHNKMKRELQRVEDSHQ  110
```

Structure of human voltage-gated sodium channel Nav1.7 in complex with auxiliary beta subunits, huwentoxin-IV and saxitoxin (Y1755 up) [Homo sapiens]
Sequence ID: **6J8G_C** Length: 215 Number of Matches: 1
Range 1: 153 to 189

Score	Expect	Method	Identities	Positives	Gaps	Frame
31.6 bits(92)	1.2()	Compositional matrix adjust.	13/40(33%)	25/40(62%)	3/40(7%)	

```
Query  257   KKKANVGAIVGGVVGGVVGAVAIALCILLIVRHINMKREQ  296
             ++ + V  IVG  VGG    +A+ + +L++V+ +  K+EQ
Sbjct  153   ERDSTVAVIVGASVGGF---LAVVILVLMVVKCVRRKKEQ  189
```

Solution structure of integrin b2 monomer tranmembrane domain in bicelle [Homo sapiens]
Sequence ID: **5ZAZ_A** Length: 52 Number of Matches: 1
Range 1: 13 to 52

Score	Expect	Method	Identities	Positives	Gaps	Frame
28.6 bits(82)	1.5()	Composition-based stats.	16/40(40%)	22/40(55%)	0/40(0%)	

```
Query  261   NVGAIVGGVVGGVVGAVAIALCILLIVRHINMKREQDRME  300
             N+ AIVGG V G+V    + L I   + H++  RE  R E
Sbjct  13    NIAAIVGGTVAGIVLIGILLLVIWKALIHLSDLREYRRFE  52
```

Structure of Tra1 subunit within the chromatin modifying complex SAGA [Komagataella pastoris]
Sequence ID: **5OEJ_B** Length: 3825 Number of Matches: 1
Range 1: 3178 to 3290

Score	Expect	Method	Identities	Positives	Gaps	Frame
32.1 bits(94)	1.8()	Composition-based stats.	25/113(22%)	45/113(39%)	1/113(0%)	

```
Query  101   EDAYSVYQLDSDTNSNSISSSDSSTESTSASSSTTSSTTSSTTSTTSSTTSSTTSSMASS  160
             ED  ++ +     N     SS+    + S   +T +    + T T+ +T  S
Sbjct  3178  EDYQAIQRQAMAVNRAEEQSSNKQDTADSVLKNTNTPQPQTRTETSGTTAESDKKPSIPP  3237

Query  161   STVQNSPESTQAAASISTSQSSSTVTSESSLTSDTLATSSTSSQS-QDATSII  212
```

```
                Q SP+ ++ A + ++ Q+ S    ESS        ++ S Q  QD   I+
Sbjct  3238  KEEQGSPQPSRPATTQASPQAQSQENGESSQKHPPEIPTTDSRQPWQDVEEIM  3290
```

The structure of an Allene Oxide Synthase reveals a novel use for a catalase fold [Plexaura homomalla]
Sequence ID: **1U5U_A** Length: 374 Number of Matches: 1
Range 1: 238 to 269

Score	Expect	Method	Identities	Positives	Gaps	Frame
30.4 bits(88)	4.5()	Compositional matrix adjust.	14/32(44%)	18/32(56%)	2/32(6%)	

```
Query  283  ILLIVRHINMKREQDRMEKEYQEAIK--PVEY  312
            I +  RH N KR  D + KEY E ++  PV Y
Sbjct  238  IWIFSRHENEKRPDDYLRKEYVERLQKGPVNY  269
```

RNA Polymerase II from Komagataella Pastoris (Type-1 crystal) [Komagataella phaffii GS115]
Sequence ID: **5X4Z_A** Length: 1743 Number of Matches: 1
Range 1: 1631 to 1739

Score	Expect	Method	Identities	Positives	Gaps	Frame
30.4 bits(88)	5.4()	Composition-based stats.	29/109(27%)	45/109(41%)	1/109(0%)	

```
Query  117   SISSSDSSTESTSASSSTTSSTTSSTTSTTSSTTSSTTSSMASSSTVQNSPESTQ-AAAS  175
             S +S   S  S   S ++    + +S   + +S   S TS    S ++ Q SP S Q +  S
Sbjct  1631  SPTSPQYSPTSPQYSPTSPQYSPTSPQYSPTSPQYSPTSPQYSPTSPQYSPTSPQYSPTS  1690

Query  176   ISTSQSSSTVTSESSLTSDTLATSSTSSQSQDATSIIYSTTFHTEGGST  224
                S +S   +  S   S T    S +S      S  YS + H+  G +
Sbjct  1691  PQYSPTSPQYSPTSPQYSPTSPQYSPTSPQYSPASPQYSPSRHSPNGES  1739
```

Taxonomy

Reports

Lineage

Organism	Blast Name	Score	Number of Hits	Description
cellular organisms			51	
.Eukaryota	eukaryotes		47	
..Opisthokonta	eukaryotes		46	
...Eumetazoa	animals		23	
....Homo sapiens	primates	36.5	19	**Homo sapiens hits**
....Plexaura homomalla	soft corals	30.4	4	**Plexaura homomalla hits**
...Komagataella pastoris	budding yeasts	32.1	1	**Komagataella pastoris hits**
...Komagataella phaffii GS115	budding yeasts	32.1	22	**Komagataella phaffii GS115 hits**
..Linanthus concinnus	eudicots	30.4	1	**Linanthus concinnus hits**
.Mycolicibacterium smegmatis MC2 155	high GC Gram+	29.5	4	**Mycolicibacterium smegmatis MC2 155 hits**

Organism

Description	Score	E value	Accession
Homo sapiens (human) [primates]			
**Structure of Coxsackievirus A10 complexed with its receptor KREMEN1 [Homo sapiens]**	36.5	0.047	**6SNW_E**
**Cryo-EM structure of mature Coxsackievirus A10 in complex with KRM1 at pH 7.4 [Homo sapiens]**	36.5	0.048	**7BZT_E**
**Cryo-EM structure of mature Coxsackievirus A10 in complex with KRM1 at pH 5.5 [Homo sapiens]**	36.5	0.048	**7BZU_E**
**Wnt modulator Kremen crystal form I at 1.90A [Homo sapiens]**	36.2	0.053	**5FWS_A**
**Wnt modulator Kremen crystal form I at 2.10A [Homo sapiens]**	36.2	0.053	**5FWT_A**
**Wnt modulator Kremen crystal form II at 2.8A [Homo sapiens]**	36.2	0.053	**5FWU_A**
**Wnt modulator Kremen crystal form III at 3.2A [Homo sapiens]**	36.2	0.053	**5FWV_A**
**Wnt modulator Kremen in complex with DKK1 (CRD2) and LRP6 (PE3PE4) [Homo sapiens]**	34.8	0.14	**5FWW_B**
**Solution NMR Structure of the serine-rich domain of hEF1 (Enhancer of filamentation 1) from Homo sapiens, Northeast Structural Genomics Consortium Target HR5554A [Homo sapiens]**	32.1	0.56	**2L81_A**
**Structure of human voltage-gated sodium channel Nav1.7 in complex with auxiliary beta subunits, huwentoxin-IV and saxitoxin (Y1755 up) [Homo sapiens]**	31.6	1.2	**6J8G_C**
**Structure of human voltage-gated sodium channel Nav1.7 in complex with auxiliary beta subunits, huwentoxin-IV and saxitoxin (Y1755 down) [Homo sapiens]**	31.6	1.2	**6J8H_C**
**Structure of human voltage-gated sodium channel Nav1.7 in complex with auxiliary beta subunits, ProTx-II and tetrodotoxin (Y1755 up) [Homo sapiens]**	31.6	1.2	**6J8I_C**
**Structure of human voltage-gated sodium channel Nav1.7 in complex with auxiliary beta subunits, ProTx-II and tetrodotoxin (Y1755 down) [Homo sapiens]**	31.6	1.2	**6J8J_C**
**Solution structure of integrin b2 monomer tranmembrane domain in bicelle [Homo sapiens]**	28.6	1.5	**5ZAZ_A**
**Solution NMR Structure of APP TMD [Homo sapiens]**	26.3	6.8	**6YHF_A**
**Solution NMR Structure of APP V44M mutant TMD [Homo sapiens]**	26.0	8.4	**6YHP_A**
**Structure of amyloid precursor protein's transmembrane domain [Homo sapiens]**	26.3	8.5	**2LLM_A**
**Dimeric structure of transmembrane domain of amyloid precursor protein in micellar environment [Homo sapiens]**	26.3	8.5	**2LOH_A**
**Dimeric structure of transmembrane domain of amyloid precursor protein in micellar environment [Homo sapiens]**	26.3	8.5	**2LOH_B**
Komagataella pastoris [budding yeasts]			
**Structure of Tra1 subunit within the chromatin modifying complex SAGA [Komagataella pastoris]**	32.1	1.8	**5OEJ_B**
Komagataella phaffii GS115 [budding yeasts]			
**Structure of SAGA bound to TBP [Komagataella phaffii GS115]**	32.1	1.8	**6TB4_L**
**Structure of SAGA bound to TBP, including Spt8 and DUB [Komagataella phaffii GS115]**	32.1	1.8	**6TBM_L**
**RNA Polymerase II from Komagataella Pastoris (Type-1 crystal) [Komagataella phaffii GS115]**	30.4	5.4	**5X4Z_A**
**RNA Polymerase II from Komagataella Pastoris (Type-1 crystal) [Komagataella phaffii GS115]**	30.4	5.4	**5X4Z_M**
**RNA Polymerase II from Komagataella Pastoris (Type-2 crystal) [Komagataella phaffii GS115]**	30.4	5.4	**5X50_A**
**RNA Polymerase II from Komagataella Pastoris (Type-3 crystal) [Komagataella phaffii GS115]**	30.4	5.4	**5X51_A**
**RNA Polymerase II from Komagataella Pastoris (Type-3 crystal) [Komagataella phaffii GS115]**	30.4	5.4	**5X51_M**
**RNA Polymerase II elongation complex bound with Spt5 KOW5 and Elf1 [Komagataella phaffii GS115]**	30.4	5.4	**5XOG_A**
**RNA Polymerase II elongation complex bound with Spt4/5 and TFIIS [Komagataella phaffii GS115]**	30.4	5.4	**5XON_A**
**RNA polymerase II elongation complex stalled at SHL(-1) of the nucleosome, with foreign DNA [Komagataella phaffii GS115]**	30.4	5.4	**6A5L_A**
**RNA polymerase II elongation complex stalled at SHL(-6) of the nucleosome [Komagataella phaffii GS115]**	30.4	5.4	**6A5O_A**
**RNA polymerase II elongation complex stalled at SHL(-5) of the nucleosome [Komagataella phaffii GS115]**	30.4	5.4	**6A5P_A**
**RNA polymerase II elongation complex stalled at SHL(-2) of the nucleosome [Komagataella phaffii GS115]**	30.4	5.4	**6A5R_A**
**RNA polymerase II elongation complex stalled at SHL(-1) of the nucleosome [Komagataella phaffii GS115]**	30.4	5.4	**6A5T_A**
**RNA polymerase II elongation complex stalled at SHL(-1) of the nucleosome, with foreign DNA, tilt conformation [Komagataella phaffii GS115]**	30.4	5.4	**6A5U_A**
**RNA polymerase II elongation complex stalled at SHL(-1) of the nucleosome, with foreign DNA (+1 position) [Komagataella phaffii GS115]**	30.4	5.4	**6INQ_A**
**RNA polymerase II elongation complex bound with Elf1 and Spt4/5, stalled at SHL(-5) of the nucleosome [Komagataella phaffii GS115]**	30.4	5.4	**6J4W_A**
**RNA polymerase II elongation complex bound with Elf1 and Spt4/5, stalled at SHL(-1) of the nucleosome (+1A) [Komagataella phaffii GS115]**	30.4	5.4	**6J4X_A**

Description	Score	E value	Accession
RNA polymerase II elongation complex bound with Elf1 and Spt4/5, stalled at SHL(-1) of the nucleosome (+1B) [Komagataella phaffii GS115]	30.4	5.4	6J4Y_A
RNA polymerase II elongation complex bound with Spt4/5 and foreign DNA, stalled at SHL(-1) of the nucleosome [Komagataella phaffii GS115]	30.4	5.4	6J4Z_A
RNA polymerase II elongation complex bound with Spt4/5 and foreign DNA, stalled at SHL(-1) of the nucleosome (tilted conformation) [Komagataella phaffii GS115]	30.4	5.4	6J50_A
RNA polymerase II elongation complex bound with Spt4/5 and foreign DNA, stalled at SHL(-1) of the nucleosome, weak Elf1 (+1 position) [Komagataella phaffii GS115]	30.4	5.4	6J51_A
Plexaura homomalla [soft corals]			
The structure of an Allene Oxide Synthase reveals a novel use for a catalase fold [Plexaura homomalla]	30.4	4.5	1U5U_A
The structure of an Allene Oxide Synthase reveals a novel use for a catalase fold [Plexaura homomalla]	30.4	4.5	1U5U_B
Allene oxide synthase 8R-lipoxygenase from Plexaura homomalla [Plexaura homomalla]	30.4	5.4	3DY5_A
Allene oxide synthase 8R-lipoxygenase from Plexaura homomalla [Plexaura homomalla]	30.4	5.4	3DY5_C
Linanthus concinnus [eudicots]			
RNA polymerase II elongation complex bound with Elf1 and Spt4/5, stalled at SHL(-1) of the nucleosome [Linanthus concinnus]	30.4	5.4	6IR9_A
Mycolicibacterium smegmatis MC2 155 [high GC Gram+]			
Allophanate Hydrolase Complex from Mycobacterium smegmatis, Msmeg0435-Msmeg0436 [Mycolicibacterium smegmatis MC2 155]	29.5	7.3	3MML_A
Allophanate Hydrolase Complex from Mycobacterium smegmatis, Msmeg0435-Msmeg0436 [Mycolicibacterium smegmatis MC2 155]	29.5	7.3	3MML_C
Allophanate Hydrolase Complex from Mycobacterium smegmatis, Msmeg0435-Msmeg0436 [Mycolicibacterium smegmatis MC2 155]	29.5	7.3	3MML_E
Allophanate Hydrolase Complex from Mycobacterium smegmatis, Msmeg0435-Msmeg0436 [Mycolicibacterium smegmatis MC2 155]	29.5	7.3	3MML_G

Taxonomy

Taxonomy	Number of hits	Number of Organisms	Description
cellular organisms	51	6	
. Eukaryota	47	5	
.. Opisthokonta	46	4	
... Eumetazoa	23	2	
.... Homo sapiens	19	1	Homo sapiens hits
.... Plexaura homomalla	4	1	Plexaura homomalla hits
... Komagataella	23	2	
.... Komagataella pastoris	1	1	Komagataella pastoris hits
.... Komagataella phaffii GS115	22	1	Komagataella phaffii GS115 hits
.. Linanthus concinnus	1	1	Linanthus concinnus hits
. Mycolicibacterium smegmatis MC2 155	4	1	Mycolicibacterium smegmatis MC2 155 hits

Top

COVID-19 is an emerging, rapidly evolving situation.
Get the latest public health information from CDC: https://www.coronavirus.gov
Get the latest research information from NIH: https://www.nih.gov/coronavirus
Find NCBI SARS-CoV-2 literature, sequence, and clinical content: https://www.ncbi.nlm.nih.gov/sars-cov-2/

BLAST ® » blastp suite » results for RID-ZGZWU4SH013

Informational Message: Frequency ratios for PSSM are all zeros, frequency ratios for BLOSUM62 will be used during traceback in composition based statistics

Informational Message: Query 'KAF1902179 Slg1p, partial.. ' (# 1): Warning: Frequency ratios for PSSM are all zeros, frequency ratios for BLOSUM62 will be used during traceback in composition based statistics

**Job Title**	gb|KAF1902179.1| ...
**RID**	ZGZWU4SH013 Search expires on 01-09 22:51 pm
**Program**	PSI-BLAST Iteration 2
**Database**	pdb
**Query ID**	KAF1902179.1
**Description**	Slg1p, partial [Saccharomyces cerevisiae] ...
**Molecule type**	amino acid
**Query Length**	378

Run PSI-Blast iteration 3

Number of sequences Run

Descriptions

Sequences with E-value BETTER than threshold

Description	Scientific Name	Max Score	Total Score	Query Cover	E value	Per. Ident	Acc. Len	Accession	Select for PSI blast	Used to build PSSM	Newly added
Wnt modulator Kremen in complex with DKK1 (CRD2) and LRP6 (PE3PE4) [Homo sapiens]	Homo sapiens	43.0	43.0	14%	5e-04	32.14%	293	5FWW_B (scored below threshold on previous iteration)	Select seq 5FWW_B for PSI blast		
Cryo-EM structure of mature Coxsackievirus A10 in complex with KRM1 at pH 7.4 [Homo sapiens]	Homo sapiens	43.0	43.0	14%	6e-04	32.14%	375	7BZT_E (scored below threshold on previous iteration)	Select seq 7BZT_E for PSI blast		
Structure of Coxsackievirus A10 complexed with its receptor KREMEN1 [Homo sapiens]	Homo sapiens	43.0	43.0	14%	6e-04	32.14%	378	6SNW_E (scored below threshold on previous iteration)	Select seq 6SNW_E for PSI blast		
Wnt modulator Kremen crystal form I at 1.90A [Homo sapiens]	Homo sapiens	43.0	43.0	14%	6e-04	32.14%	406	5FWS_A (scored below threshold on previous iteration)	Select seq 5FWS_A for PSI blast		

Run PSI-BLAST Iteration 3 with max number of sequences Run

Sequences with E-value WORSE than threshold

Description	Scientific Name	Max Score	Total Score	Query Cover	E value	Per. Ident	Acc. Len	Accession	Select for PSI blast	Used to build PSSM	Newly added
eIF2B:eIF2 complex, phosphorylated on eIF2 alpha serine 52 [Saccharomyces cerevisiae S288C]	Saccharomyces cerevisiae S288C	39.9	75.1	26%	0.006	28.18%	651	6I3M_C (scored below threshold on previous iteration)	Select seq 6I3M_C for PSI blast		
EBOV GP in complex with variable Fab domains of IgGs c2G4 and c13C6 [Ebola virus - Mayinga, Zaire, 1976]	Ebola virus - Mayinga, Zaire, 1976	38.3	38.3	44%	0.019	20.35%	469	5KEL_A (scored below threshold on previous iteration)	Select seq 5KEL_A for PSI blast		
PROTON NUCLEAR MAGNETIC RESONANCE AND DISTANCE GEOMETRY(SLASH)SIMULATED ANNEALING STUDIES ON THE VARIANT-1 NEUROTOXIN FROM THE NEW WORLD SCORPION CENTRUROIDES SCULPTURATUS EWING [Centruroides sculpturatus]	Centruroides sculpturatus	34.5	34.5	10%	0.027	41.46%	65	1VNA_A (scored below threshold on previous iteration)	Select seq 1VNA_A for PSI blast		
Bacteriophage T4 isometric capsid [Escherichia virus T4]	Escherichia virus T4	37.6	37.6	41%	0.028	23.93%	376	5VF3_Z (scored below threshold on previous iteration)	Select seq 5VF3_Z for PSI blast		
Structure of Tra1 subunit within the chromatin modifying complex SAGA [Komagataella pastoris]	Komagataella pastoris	35.6	35.6	26%	0.17	24.51%	3825	5OEJ_B (scored below threshold on previous iteration)	Select seq 5OEJ_B for PSI blast		
The solution NMR structure of the transmembrane C-terminal domain of the amyloid precursor protein (C99) [Homo sapiens]	Homo sapiens	32.9	32.9	23%	0.24	25.74%	122	2LP1_A (scored below threshold on previous iteration)	Select seq 2LP1_A for PSI blast		
Solution structure of integrin b2 monomer tranmembrane domain in bicelle [Homo sapiens]	Homo sapiens	31.0	31.0	10%	0.35	40.00%	52	5ZAZ_A (scored below threshold on previous iteration)	Select seq 5ZAZ_A for PSI blast		
Three-Dimensional Structure Of a Single Chain Fv Fragment Complexed With The peptide GCN4(7P-14P) [Mus musculus]	Mus musculus	32.6	32.6	26%	0.42	29.25%	135	1P4B_L (scored below threshold on previous iteration)	Select seq 1P4B_L for PSI blast		
Crystal structure of glycopeptide 22 in complex with scFv-SM3 [Mus musculus]	Mus musculus	33.3	33.3	26%	0.44	28.30%	244	5FXC_H (scored below threshold on previous iteration)	Select seq 5FXC_H for PSI blast		
Solution NMR Structure of the serine-rich domain of hEF1 (Enhancer of filamentation 1) from Homo sapiens, Northeast Structural Genomics Consortium Target HR5554A [Homo sapiens]	Homo sapiens	32.9	32.9	8%	0.48	46.88%	176	2L81_A (scored below threshold on previous iteration)	Select seq 2L81_A for PSI blast		

Description	Scientific Name	Max Score	Total Score	Query Cover	E value	Per. Ident	Acc. Len	Accession	Select for PSI blast	Used to build PSSM	Newly added
Application of anti-helix antibodies in protein structure determination (9014-1P4B) [Mus musculus]	Mus musculus	32.2	32.2	26%	0.48	29.25%	115	6K65_L (scored below threshold on previous iteration)	Select seq 6K65_L for PSI blast		
Crystal structure of scFv-SM3 in complex with APD-SGalNAc-RP [Mus musculus]	Mus musculus	32.9	32.9	26%	0.60	28.30%	244	5A2I_H (scored below threshold on previous iteration)	Select seq 5A2I_H for PSI blast		
Solution NMR Structure of APP TMD [Homo sapiens]	Homo sapiens	29.5	29.5	7%	0.65	44.44%	30	6YHF_A (scored below threshold on previous iteration)	Select seq 6YHF_A for PSI blast		
Solution NMR Structure of APP V44M mutant TMD [Homo sapiens]	Homo sapiens	29.1	29.1	7%	0.85	44.44%	30	6YHP_A (scored below threshold on previous iteration)	Select seq 6YHP_A for PSI blast		
Structure of amyloid precursor protein's transmembrane domain [Homo sapiens]	Homo sapiens	29.5	29.5	7%	0.86	44.44%	43	2LLM_A (scored below threshold on previous iteration)	Select seq 2LLM_A for PSI blast		
Candida albicans PKh Kinase Domain [Candida albicans SC5314]	Candida albicans SC5314	32.6	32.6	22%	1.1	29.41%	974	4C0T_A (scored below threshold on previous iteration)	Select seq 4C0T_A for PSI blast		
Recognition of the Amyloid Precursor Protein by Human gamma-secretase [Homo sapiens]	Homo sapiens	29.5	29.5	7%	3.0	44.44%	104	6IYC_E (scored below threshold on previous iteration)	Select seq 6IYC_E for PSI blast		
Solution NMR structure of transmembrane domain of amyloid precursor protein WT [Homo sapiens]	Homo sapiens	27.6	27.6	6%	3.4	45.83%	31	2LZ3_A (scored below threshold on previous iteration)	Select seq 2LZ3_A for PSI blast		
Solution NMR Structure of APP I45T mutant TMD [Homo sapiens]	Homo sapiens	27.6	27.6	7%	3.6	40.74%	30	6YHX_A (scored below threshold on previous iteration)	Select seq 6YHX_A for PSI blast		
Cryo-EM structure of a human spliceosome activated for step 2 of splicing (C* complex) [Homo sapiens]	Homo sapiens	30.6	30.6	32%	4.9	25.52%	2752	5MQF_S (scored below threshold on previous iteration)	Select seq 5MQF_S for PSI blast		
Crystal structure of the ligand binding region of staphylococcal adhesion SraP [Staphylococcus aureus subsp. aureus NCTC 8325]	Staphylococcus aureus subsp. aureus NCTC 8325	30.3	30.3	37%	6.0	18.88%	541	4M00_A (scored below threshold on previous iteration)	Select seq 4M00_A for PSI blast		

Graphic Summary

Distribution of the top 26 Blast Hits on 25 subject sequences

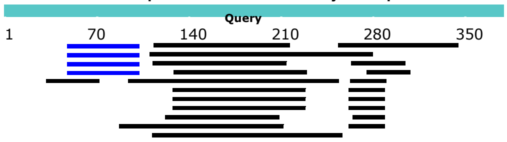

Alignments

Alignment view Pairwise ☐ CDS feature Restore defaults

Wnt modulator Kremen in complex with DKK1 (CRD2) and LRP6 (PE3PE4) [Homo sapiens]
Sequence ID: **5FWW_B** Length: 293 Number of Matches: 1
Range 1: 118 to 172

Score	Expect	Method	Identities	Positives	Gaps	Frame
43.0 bits(99)	5e-04()	Composition-based stats.	18/56(32%)	31/56(55%)	4/56(7%)	

```
Query  49   CNSECSAKGASYFALYNHSECYCGDTNP---SGSESTSSSCNTYCFGYSSEMCGGE  101
            C S C ++    +   + +    C+CG+ NP       E+ S+ CN+ CFG  ++ CGG+
Sbjct  118  CISFCRSQRFKFAGMESGYACFCGN-NPDYWKYGEAASTECNSVCFGDHTQPCGGD  172
```

Cryo-EM structure of mature Coxsackievirus A10 in complex with KRM1 at pH 7.4 [Homo sapiens]
Sequence ID: **7BZT_E** Length: 375 Number of Matches: 1
Range 1: 127 to 181

Score	Expect	Method	Identities	Positives	Gaps	Frame
43.0 bits(99)	6e-04()	Composition-based stats.	18/56(32%)	31/56(55%)	4/56(7%)	

```
Query  49   CNSECSAKGASYFALYNHSECYCGDTNP---SGSESTSSSCNTYCFGYSSEMCGGE  101
            C S C ++    +   + +    C+CG+ NP       E+ S+ CN+ CFG  ++ CGG+
Sbjct  127  CISFCRSQRFKFAGMESGYACFCGN-NPDYWKYGEAASTECNSVCFGDHTQPCGGD  181
```

Structure of Coxsackievirus A10 complexed with its receptor KREMEN1 [Homo sapiens]
Sequence ID: **6SNW_E** Length: 378 Number of Matches: 1
Range 1: 130 to 184

Score	Expect	Method	Identities	Positives	Gaps	Frame
43.0 bits(99)	6e-04()	Composition-based stats.	18/56(32%)	31/56(55%)	4/56(7%)	

```
Query  49   CNSECSAKGASYFALYNHSECYCGDTNP---SGSESTSSSCNTYCFGYSSEMCGGE  101
            C S C ++    +   + +    C+CG+ NP       E+ S+ CN+ CFG  ++ CGG+
Sbjct  130  CISFCRSQRFKFAGMESGYACFCGN-NPDYWKYGEAASTECNSVCFGDHTQPCGGD  184
```

Wnt modulator Kremen crystal form I at 1.90A [Homo sapiens]
Sequence ID: **5FWS_A** Length: 406 Number of Matches: 1
Range 1: 158 to 212

Score	Expect	Method	Identities	Positives	Gaps	Frame
43.0 bits(99)	6e-04()	Composition-based stats.	18/56(32%)	31/56(55%)	4/56(7%)	

```
Query  49   CNSECSAKGASYFALYNHSECYCGDTNP---SGSESTSSSCNTYCFGYSSEMCGGE  101
```

```
            C S C ++   +  + +   C+CG+ NP      E+ S+ CN+ CFG  ++ CGG+
Sbjct  158  CISFCRSQRFKFAGMESGYACFCGN-NPDYWKYGEAASTECNSVCFGDHTQPCGGD  212
```

eIF2B:eIF2 complex, phosphorylated on eIF2 alpha serine 52. [Saccharomyces cerevisiae S288C]
Sequence ID: **6I3M_C** Length: 651 Number of Matches: 2
Range 1: 152 to 259

Score	Expect	Method	Identities	Positives	Gaps	Frame
39.9 bits(91)	0.006()	Composition-based stats.	31/110(28%)	58/110(52%)	10/110(9%)	

```
Query  113  TNSNSISSSDSSTESTSASSSTTSSTTSSTTSTTSSTTSSTTSSMASSSTVQNSPESTQA  172
            + SN +++S       +ASS+ ++S  +STT+T  +++++ ++  +S+ST  N+P + Q
Sbjct  152  SGSNVLTASSLMPVGPNASSTVSASAPASTTTTLPASSAALSAGTSSAST--NTPTAIQQ  209

Query  173  A-ASISTSQSSSTVTSESSLTSDTLATSSTSS-------QSQDATSIIYS  214
              AS + S  + T+ S S    +       SS       QS D +S+I S
Sbjct  210  EIASSNASDVAKTLASISLEAGEFNVIPGISSVIPTVLEQSFDNSSLISS  259
```

Range 2: 147 to 227

Score	Expect	Method	Identities	Positives	Gaps	Frame
35.3 bits(79)	0.16()	Composition-based stats.	23/83(28%)	45/83(54%)	2/83(2%)	

```
Query  118  ISSSDSSTESTSASSSTTSSTTSSTTSTTSSTTSSTTSSMASSSTVQNSPESTQAAASIS  177
            ++S+ S +   +ASS       +S+T + S+  S+TT+  ASS+ +  S  ++ A+ +
Sbjct  147  VASALSGSNVLTASSLMPVGPNASSTVSASAPASTTTTLPASSAAL--SAGTSSASTNTP  204

Query  178  TSQSSSTVTSESSLTSDTLATSS  200
            T+      +S +S  + TLA+ S
Sbjct  205  TAIQQEIASSNASDVAKTLASIS  227
```

EBOV GP in complex with variable Fab domains of IgGs c2G4 and c13C6 [Ebola virus - Mayinga, Zaire, 1976]
Sequence ID: **5KEL_A** Length: 469 Number of Matches: 1
Range 1: 301 to 461

Score	Expect	Method	Identities	Positives	Gaps	Frame
38.3 bits(87)	0.019()	Composition-based stats.	35/172(20%)	71/172(41%)	15/172(8%)	

```
Query  110  DSDTNSNSISSSDSSTESTSASSSTTSSTTS--STTSTTSSTTSSTTSSMASSSTVQNSP  167
            ++ T  + I +S++S+       S    +  S  +T +T S++  S T+     ++  N+P
Sbjct  301  NTTTEDHKIMASENSSAMVQVHSQGREAAVSHLTTLATISTSPQSLTTKPGPDNSTHNTP  360

Query  168  ESTQAAASISTSQSSSTVTSESSLTSDTLATSSTSSQSQDATSIIYSTTFHTEGGSTIFV  227
                  +  +  +     T   S  SDT + ++ +   +   +            ST F+
Sbjct  361  VYKLDISEATQVEQHHRRTDNDSTASDTPSATTAAGPPKAENT--------NTSKSTDFL  412

Query  228  TNTITASAQNSGSATGTAGSDSTSGSKTHKKKANVG--AIVGGVVGGVVGAV  277
                T S QN      TAG+++T    T ++ A+ G   ++   + GV G +
Sbjct  413  DPATTTSPQNHSE---TAGNNNTHHQDTGEESASSGKLGLITNTIAGVAGLI  461
```

PROTON NUCLEAR MAGNETIC RESONANCE AND DISTANCE GEOMETRY(SLASH)SIMULATED ANNEALING STUDIES ON THE VARIANT-1 NEUROTOXIN FROM THE NEW WORLD SCORPION CENTRUROIDES SCULPTURATUS EWING [Centruroides sculpturatus]
Sequence ID: **1VNA_A** Length: 65 Number of Matches: 1
Range 1: 9 to 48

Score	Expect	Method	Identities	Positives	Gaps	Frame
34.5 bits(77)	0.027()	Composition-based stats.	17/41(41%)	22/41(53%)	3/41(7%)	

```
Query  33   SDFSKADSYNWQSSSHCNSECSAK--GASYFALYNHSECYC  71
            SD  K D +    + HCN+EC AK  G SY   Y  + C+C
Sbjct  9    SDGCKYDCFWLGKNEHCNTECKAKNQGGSYGYCYAFA-CWC  48
```

Bacteriophage T4 isometric capsid [Escherichia virus T4]
Sequence ID: **5VF3_Z** Length: 376 Number of Matches: 1
Range 1: 122 to 281

Score	Expect	Method	Identities	Positives	Gaps	Frame
37.6 bits(85)	0.028()	Composition-based stats.	39/163(24%)	68/163(41%)	8/163(4%)	

```
Query  94   SSEMCGGEDAYSVYQLDSDTNSNSISSSDSSTESTSASSSTTSSTTSSTTSTTSSTTSST  153
            +S+  G    Y  Y  DS     + +S+ S T +TS           + T   + + +S
Sbjct  122  ASQPDGASATYQWYVDDSQVGGET-NSTFSYTPTTSGVKRIKCVAQVTATDYDALSVTSN  180

Query  154  TSSMASSSTVQNSPESTQAAASISTSQSSSTVTSESSLTSDTLATSSTSSQSQDATSI--  211
              S+  +    N P+ T    SI+  Q +S  T  +++T        T S  +D++ +
Sbjct  181  EVSLTVNKKTMN-PQVTLTPPSINVQQDASA-TFTANVTGAPEEAQITYSWKKDSSPVEG  238

Query  212  ---IYSTTFHTEGGSTIFVTNTITASAQNSGSATGTAGSDSTS  251
               +Y+    + G  TI VT T+TA+  N  + T T     T+
Sbjct  239  STNVYTVDTSSVGSQTIEVTATVTAADYNPVTVTKTGNVTVTA  281
```

Structure of Tra1 subunit within the chromatin modifying complex SAGA [Komagataella pastoris]
Sequence ID: **5OEJ_B** Length: 3825 Number of Matches: 1
Range 1: 3195 to 3290

Score	Expect	Method	Identities	Positives	Gaps	Frame
35.6 bits(80)	0.17()	Composition-based stats.	25/102(25%)	45/102(44%)	7/102(6%)	

```
Query  112   DTNSNSISSSDSSTESTSASSSTTSSTTSSTTSTTSSTTSSTTSSMASSSTVQNSPESTQ  171
             + +SN   ++DS  ++T+     T + TS TT+ +    S            Q SP+ ++
Sbjct  3195  EQSSNKQDTADSVLKNTNTPQPQTRTETSGTTAESDKKPS------IPPKEEQGSPQPSR  3248

Query  172   AAASISTSQSSSTVTSESSLTSDTLATSSTSSQS-QDATSII  212
              A + ++ Q+ S    ESS        ++ S Q  QD   I+
Sbjct  3249  PATTQASPQAQSQENGESSQKHPPEIPTTDSRQPWQDVEEIM  3290
```

The solution NMR structure of the transmembrane C-terminal domain of the amyloid precursor protein (C99) [Homo sapiens]
Sequence ID: **2LP1_A** Length: 122 Number of Matches: 1
Range 1: 9 to 103

Score	Expect	Method	Identities	Positives	Gaps	Frame
32.9 bits(73)	0.24()	Composition-based stats.	26/101(26%)	46/101(45%)	17/101(16%)	

```
Query  251  SGSKTHKKK---------ANVGAIVGGVVGGVVGAVAIALCILLIVRHINMKREQDRMEK  301
            SG + H +K         +N GAI+G +VGGVV A  I + ++++      K++   +
Sbjct  9    SGYEVHHQKLVFFAEDVGSNKGAIIGLMVGGVVIATVIVITLVML-----KKKQYTSIHH  63

Query  302  EYQEAIKPVEYPDKLYASSFSSN--HGPSSGSFEEEHTKGQ  340
               E    V  P++ + S    N    P+   FE+   +G+
Sbjct  64   GVVEVDAAVT-PEERHLSKMQQNGYENPTYKFFEQMQNQGR  103
```

Solution structure of integrin b2 monomer tranmembrane domain in bicelle [Homo sapiens]
Sequence ID: **5ZAZ_A** Length: 52 Number of Matches: 1
Range 1: 13 to 52

Score	Expect	Method	Identities	Positives	Gaps	Frame
31.0 bits(68)	0.35()	Composition-based stats.	16/40(40%)	22/40(55%)	0/40(0%)	

```
Query  261  NVGAIVGGVVGGVVGAVAIALCILLIVRHINMKREQDRME  300
```

```
            N+ AIVGG V G+V    + L I   + H++  RE  R E
Sbjct  13   NIAAIVGGTVAGIVLIGILLLVIWKALIHLSDLREYRRFE  52
```

Three-Dimensional Structure Of a Single Chain Fv Fragment Complexed With The peptide GCN4(7P-14P). [Mus musculus]
Sequence ID: **1P4B_L** Length: 135 Number of Matches: 1
Range 1: 10 to 113

Score	Expect	Method	Identities	Positives	Gaps	Frame
32.6 bits(72)	0.42()	Composition-based stats.	31/106(29%)	49/106(46%)	8/106(7%)	

```
Query  128  TSASSSTTSSTTSSTTSTTSSTTSSTTSSMASSSTVQNSPESTQAAASISTSQSSSTVTS  187
            T  S+ TTS   + T +  SST + TTS+ A  S VQ  P+         T+  +  V +
Sbjct  10   TQESALTTSPGETVTLTCRSSTGAVTTSNYA--SWVQEKPDHLFTGLIGGTNNRAPGVPA  67

Query  188  E--SSLTSDTLATSSTSSQSQDAT----SIIYSTTFHTEGGSTIFV  227
                SL  D  A + T +Q++D      ++ YS  +   GG+ + V
Sbjct  68   RFSGSLIGDKAALTITGAQTEDEAIYFCALWYSNHWVFGGGTKLTV  113
```

Crystal structure of glycopeptide 22 in complex with scFv-SM3 [Mus musculus]
Sequence ID: **5FXC_H** Length: 244 Number of Matches: 1
Range 1: 139 to 242

Score	Expect	Method	Identities	Positives	Gaps	Frame
33.3 bits(74)	0.44()	Composition-based stats.	30/106(28%)	49/106(46%)	8/106(7%)	

```
Query  128  TSASSSTTSSTTSSTTSTTSSTTSSTTSSMASSSTVQNSPESTQAAASISTSQSSSTVTS  187
            T  S+ TTS   + T +  SST + TTS+ A  + VQ  P+         T+  +  V +
Sbjct  139  TQESALTTSPGETVTLTCRSSTGAVTTSNYA--NWVQEKPDHLFTGLIGGTNNRAPGVPA  196

Query  188  E--SSLTSDTLATSSTSSQSQDAT----SIIYSTTFHTEGGSTIFV  227
                SL  D  A + T +Q++D      ++ YS  +   GG+ + V
Sbjct  197  RFSGSLIGDKAALTITGAQTEDEAIYFCALWYSNHWVFGGGTKLTV  242
```

Solution NMR Structure of the serine-rich domain of hEF1 (Enhancer of filamentation 1) from Homo sapiens, Northeast Structural Genomics Consortium Target HR5554A [Homo sapiens]
Sequence ID: **2L81_A** Length: 176 Number of Matches: 1
Range 1: 79 to 110

Score	Expect	Method	Identities	Positives	Gaps	Frame
32.9 bits(73)	0.48()	Composition-based stats.	15/32(47%)	20/32(62%)	0/32(0%)	

```
Query  273  VVGAVAIALCILLIVRHINMKREQDRMEKEYQ  304
            V GAVA A C+  ++ H  MKRE  R+E  +Q
Sbjct  79   VKGAVANAACLPELILHNKMKRELQRVEDSHQ  110
```

Application of anti-helix antibodies in protein structure determination (9014-1P4B) [Mus musculus]
Sequence ID: **6K65_L** Length: 115 Number of Matches: 1
Range 1: 5 to 108

Score	Expect	Method	Identities	Positives	Gaps	Frame
32.2 bits(71)	0.48()	Composition-based stats.	31/106(29%)	49/106(46%)	8/106(7%)	

```
Query  128  TSASSSTTSSTTSSTTSTTSSTTSSTTSSMASSSTVQNSPESTQAAASISTSQSSSTVTS  187
            T  S+ TTS   + T +  SST + TTS+ A  S VQ  P+         T+  +  V +
Sbjct  5    TQESALTTSPGETVTLTCRSSTGAVTTSNYA--SWVQEKPDHLFTGLIGGTNNRAPGVPA  62

Query  188  E--SSLTSDTLATSSTSSQSQDAT----SIIYSTTFHTEGGSTIFV  227
                SL  D  A + T +Q++D      ++ YS  +   GG+ + V
Sbjct  63   RFSGSLIGDKAALTITGAQTEDEAIYFCALWYSNHWVFGGGTKLTV  108
```

Crystal structure of scFv-SM3 in complex with APD-SGalNAc-RP [Mus musculus]
Sequence ID: **5A2I_H** Length: 244 Number of Matches: 1
Range 1: 139 to 242

Score	Expect	Method	Identities	Positives	Gaps	Frame
32.9 bits(73)	0.60()	Composition-based stats.	30/106(28%)	49/106(46%)	8/106(7%)	

```
Query  128  TSASSSTTSSTTSSTTSTTSSTTSSTTSSMASSSTVQNSPESTQAAASISTSQSSSTVTS  187
            T  S+ TTS   + T +  SST + TTS+ A  + VQ  P+         T+  +  V +
Sbjct  139  TQESALTTSPGETVTLTCRSSTGAVTTSNYA--NWVQEKPDHLFTGLIGGTNNRAPGVPA  196

Query  188  E--SSLTSDTLATSSTSSQSQDAT----SIIYSTTFHTEGGSTIFV  227
                SL  D  A + T +Q++D      ++ YS  +   GG+ + V
Sbjct  197  RFSGSLIGDKAALTITGAQTEDEAIYFCALWYSNHWVFGGGTKLTV  242
```

Solution NMR Structure of APP TMD [Homo sapiens]
Sequence ID: **6YHF_A** Length: 30 Number of Matches: 1
Range 1: 1 to 27

Score	Expect	Method	Identities	Positives	Gaps	Frame
29.5 bits(64)	0.65()	Composition-based stats.	12/27(44%)	20/27(74%)	0/27(0%)	

```
Query  260  ANVGAIVGGVVGGVVGAVAIALCILLI  286
            +N GAI+G +VGGVV A  I + ++++
Sbjct  1    SNKGAIIGLMVGGVVIATVIVITLVML  27
```

Solution NMR Structure of APP V44M mutant TMD [Homo sapiens]
Sequence ID: **6YHP_A** Length: 30 Number of Matches: 1
Range 1: 1 to 27

Score	Expect	Method	Identities	Positives	Gaps	Frame
29.1 bits(63)	0.85()	Composition-based stats.	12/27(44%)	20/27(74%)	0/27(0%)	

```
Query  260  ANVGAIVGGVVGGVVGAVAIALCILLI  286
            +N GAI+G +VGGVV A  I + ++++
Sbjct  1    SNKGAIIGLMVGGVVIATMIVITLVML  27
```

Structure of amyloid precursor protein's transmembrane domain [Homo sapiens]
Sequence ID: **2LLM_A** Length: 43 Number of Matches: 1
Range 1: 14 to 40

Score	Expect	Method	Identities	Positives	Gaps	Frame
29.5 bits(64)	0.86()	Composition-based stats.	12/27(44%)	20/27(74%)	0/27(0%)	

```
Query  260  ANVGAIVGGVVGGVVGAVAIALCILLI  286
            +N GAI+G +VGGVV A  I + ++++
Sbjct  14   SNKGAIIGLMVGGVVIATVIVITLVML  40
```

Candida albicans PKh Kinase Domain [Candida albicans SC5314]
Sequence ID: **4C0T_A** Length: 974 Number of Matches: 1
Range 1: 598 to 671

Score	Expect	Method	Identities	Positives	Gaps	Frame

32.6 bits(72) 1.1() Composition-based stats. 25/85(29%) 36/85(42%) 11/85(12%)

```
Query  123   SSTESTSASSSTTSSTTSSTTSTTSSTTSSTTSSMASSSTVQNSPESTQAAASISTSQSS  182
             SS+     SS T     S  T    ST   + +S+A+   V N P         +T+Q+S
Sbjct  598   SSSNGHKGSSPTPEKEPSPATINNKSTEKVSAASVAA--YVLNKP---------ATNQNS  646

Query  183   STVTSESSLTSDTLATSSTSSQSQD  207
             ST    S  +S++  T   S   QD
Sbjct  647   STSEDSSKRSSNSNETRKLSYSQQD  671
```

Recognition of the Amyloid Precursor Protein by Human gamma-secretase [Homo sapiens]
Sequence ID: **6IYC_E** Length: 104 Number of Matches: 1
Range 1: 11 to 37

Score	Expect	Method	Identities	Positives	Gaps	Frame
29.5 bits(64)	3.0()	Composition-based stats.	12/27(44%)	20/27(74%)	0/27(0%)	

```
Query  260   ANVGAIVGGVVGGVVGAVAIALCILLI  286
             +N GAI+G +VGGVV A  I + ++++
Sbjct  11    SNKGAIIGLMVGGVVIATVIVITLVML  37
```

Solution NMR structure of transmembrane domain of amyloid precursor protein WT [Homo sapiens]
Sequence ID: **2LZ3_A** Length: 31 Number of Matches: 1
Range 1: 5 to 28

Score	Expect	Method	Identities	Positives	Gaps	Frame
27.6 bits(59)	3.4()	Composition-based stats.	11/24(46%)	18/24(75%)	0/24(0%)	

```
Query  263   GAIVGGVVGGVVGAVAIALCILLI  286
             GAI+G +VGGVV A  I + ++++
Sbjct  5     GAIIGLMVGGVVIATVIVITLVML  28
```

Solution NMR Structure of APP I45T mutant TMD [Homo sapiens]
Sequence ID: **6YHX_A** Length: 30 Number of Matches: 1
Range 1: 1 to 27

Score	Expect	Method	Identities	Positives	Gaps	Frame
27.6 bits(59)	3.6()	Composition-based stats.	11/27(41%)	19/27(70%)	0/27(0%)	

```
Query  260   ANVGAIVGGVVGGVVGAVAIALCILLI  286
             +N GAI+G +VGGVV A    + ++++
Sbjct  1     SNKGAIIGLMVGGVVIATVTVITLVML  27
```

Cryo-EM structure of a human spliceosome activated for step 2 of splicing (C* complex) [Homo sapiens]
Sequence ID: **5MQF_S** Length: 2752 Number of Matches: 1
Range 1: 1034 to 1178

Score	Expect	Method	Identities	Positives	Gaps	Frame
30.6 bits(67)	4.9()	Composition-based stats.	37/145(26%)	56/145(38%)	22/145(15%)	

```
Query  88    TYCFGYSSEMCGGEDAYSVYQLD------------SDTNSNSISSSDSSTESTSASSSTT  135
             + C G  S    GE  + V  L             SDT+S  +  S S + S  + S T+
Sbjct  1034  SLCAGVKSSTPPGESYFGVSSLQLKGQSQTSPDHRSDTSSPEVRQSHSESPSLQSKSQTS  1093

Query  136   -----SSTTSSTTSTTSSTTSSTTSSMASSSTVQNSPESTQAAASISTSQSSSTVTSESS  190
                  S ++S  T   S +         S+S +  S  S + +   S S S  TV S S
Sbjct  1094  PKGGRSRSSSPVTELASRSPIRQDRGEFSASPMLKSGMSPEQSRFQSDSSSYPTVDSNSL  1153

Query  191   LTSDTLATSSTSSQ-----SQDATS  210
             L    L T+ +  +      +DAT+
Sbjct  1154  LGQSRLETAESKEKMALPPQEDATA  1178
```

Crystal structure of the ligand binding region of staphylococcal adhesion SraP [Staphylococcus aureus subsp. aureus NCTC 8325]
Sequence ID: **4M00_A** Length: 541 Number of Matches: 1
Range 1: 388 to 530

Score	Expect	Method	Identities	Positives	Gaps	Frame
30.3 bits(66)	6.0()	Composition-based stats.	27/143(19%)	63/143(44%)	0/143(0%)	

```
Query  113   TNSNSISSSDSSTESTSASSSTTSSTTSSTTSTTSSTTSSTTSSMASSSTVQNSPESTQA  172
             T +++ + + ++T +   S  +  S T+S   T +    ST + +++      S  +
Sbjct  388   TTTDNGTGTVTNTVTGLPSGLSYDSATNSIIGTPTKIGQSTVTVVSTDQANNKSTTTFTI  447

Query  173   AASISTSQSSSTVTSESSLTSDTLATSSTSSQSQDATSIIYSTTFHTEGGSTIFVTNTIT  232
                 +T+ + + +  +SS     ++    ++Q     ++ + T    G +      NTI+
Sbjct  448   NVVDTTAPTVTPIGDQSSEVYSPISPIKIATQDNSGNAVTNTVTGLPSGLTFDSTNNTIS  507

Query  233   ASAQNSGSATGTAGSDSTSGSKT  255
              +  N G++T +  S   SG+KT
Sbjct  508   GTPTNIGTSTISIVSTDASGNKT  530
```

Taxonomy

Reports

Lineage

Organism	Blast Name	Score	Number of Hits	Description
root			71	
.cellular organisms			67	
..Opisthokonta	eukaryotes		66	
...Bilateria	animals		46	
....Euarchontoglires	placentals		44	
.....Homo sapiens	primates	43.0	30	**Homo sapiens hits**
.....Mus musculus	rodents	32.6	14	**Mus musculus hits**
....Centruroides sculpturatus	scorpions	34.5	2	**Centruroides sculpturatus hits**
...Saccharomyces cerevisiae S288C	budding yeasts	39.9	10	**Saccharomyces cerevisiae S288C hits**
...Saccharomyces cerevisiae	budding yeasts	39.9	6	**Saccharomyces cerevisiae hits**
...Komagataella pastoris	budding yeasts	35.6	1	**Komagataella pastoris hits**
...Komagataella phaffii GS115	budding yeasts	35.6	2	**Komagataella phaffii GS115 hits**
...Candida albicans SC5314	budding yeasts	32.6	1	**Candida albicans SC5314 hits**
..Staphylococcus aureus subsp. aureus NCTC 8325	firmicutes	30.3	1	**Staphylococcus aureus subsp. aureus NCTC 8325 hits**
.Ebola virus - Mayinga, Zaire, 1976	viruses	38.3	3	**Ebola virus - Mayinga, Zaire, 1976 hits**
.Escherichia virus T4	viruses	37.6	1	**Escherichia virus T4 hits**

Organism

Description	Score	E	Accession

		value	
Homo sapiens (human) [primates]			
Wnt modulator Kremen in complex with DKK1 (CRD2) and LRP6 (PE3PE4) [Homo sapiens]	43.0	5e-04	5FWW_B
Cryo-EM structure of mature Coxsackievirus A10 in complex with KRM1 at pH 7.4 [Homo sapiens]	43.0	6e-04	7BZT_E
Cryo-EM structure of mature Coxsackievirus A10 in complex with KRM1 at pH 5.5 [Homo sapiens]	43.0	6e-04	7BZU_E
Structure of Coxsackievirus A10 complexed with its receptor KREMEN1 [Homo sapiens]	43.0	6e-04	6SNW_E
Wnt modulator Kremen crystal form I at 1.90A [Homo sapiens]	43.0	6e-04	5FWS_A
Wnt modulator Kremen crystal form I at 2.10A [Homo sapiens]	43.0	6e-04	5FWT_A
Wnt modulator Kremen crystal form II at 2.8A [Homo sapiens]	43.0	6e-04	5FWU_A
Wnt modulator Kremen crystal form III at 3.2A [Homo sapiens]	43.0	6e-04	5FWV_A
The solution NMR structure of the transmembrane C-terminal domain of the amyloid precursor protein (C99) [Homo sapiens]	32.9	0.24	2LP1_A
Solution structure of integrin b2 monomer tranmembrane domain in bicelle [Homo sapiens]	31.0	0.35	5ZAZ_A
Solution NMR Structure of the serine-rich domain of hEF1 (Enhancer of filamentation 1) from Homo sapiens, Northeast Structural Genomics Consortium Target HR5554A [Homo sapiens]	32.9	0.48	2L81_A
Solution NMR Structure of APP TMD [Homo sapiens]	29.5	0.65	6YHF_A
Solution NMR Structure of APP V44M mutant TMD [Homo sapiens]	29.1	0.85	6YHP_A
Structure of amyloid precursor protein's transmembrane domain [Homo sapiens]	29.5	0.86	2LLM_A
Dimeric structure of transmembrane domain of amyloid precursor protein in micellar environment [Homo sapiens]	29.5	0.86	2LOH_A
Dimeric structure of transmembrane domain of amyloid precursor protein in micellar environment [Homo sapiens]	29.5	0.86	2LOH_B
Recognition of the Amyloid Precursor Protein by Human gamma-secretase [Homo sapiens]	29.5	3.0	6IYC_E
Solution NMR structure of transmembrane domain of amyloid precursor protein WT [Homo sapiens]	27.6	3.4	2LZ3_A
Solution NMR structure of transmembrane domain of amyloid precursor protein WT [Homo sapiens]	27.6	3.4	2LZ3_B
Solution NMR Structure of APP I45T mutant TMD [Homo sapiens]	27.6	3.6	6YHX_A
Cryo-EM structure of a human spliceosome activated for step 2 of splicing (C* complex) [Homo sapiens]	30.6	4.9	5MQF_S
Cryo-EM structure of the human spliceosome just prior to exon ligation at 3.6 angstrom [Homo sapiens]	30.6	4.9	5XJC_U
The Cryo-EM Structure of Human Catalytic Step I Spliceosome (C complex) at 4.1 angstrom resolution [Homo sapiens]	30.6	4.9	5YZG_U
cryo-EM structure of a human activated spliceosome (mature Bact) at 5.1 angstrom. [Homo sapiens]	30.6	4.9	5Z56_U
Cryo-EM structure of the human activated spliceosome (late Bact) at 6.5 angstrom [Homo sapiens]	30.6	4.9	5Z57_U
human Bact spliceosome core structure [Homo sapiens]	30.6	4.9	6FF4_S
human Bact spliceosome core structure [Homo sapiens]	30.6	4.9	6FF7_S
Cryo-EM structure of a human post-catalytic spliceosome (P complex) at 3.0 angstrom [Homo sapiens]	30.6	4.9	6ICZ_U
Human C Complex Spliceosome - High-resolution CORE [Homo sapiens]	30.6	4.9	6ZYM_S
Human C Complex Spliceosome - Medium-resolution PERIPHERY [Homo sapiens]	30.6	4.9	7A5P_S
Saccharomyces cerevisiae S288C [budding yeasts]			
eIF2B:eIF2 complex, phosphorylated on eIF2 alpha serine 52. [Saccharomyces cerevisiae S288C]	39.9	0.006	6I3M_C
eIF2B:eIF2 complex, phosphorylated on eIF2 alpha serine 52. [Saccharomyces cerevisiae S288C]	39.9	0.006	6I3M_D
Structure of eIF2B-eIF2 (phosphorylated at Ser51) complex (model 1) [Saccharomyces cerevisiae S288C]	39.9	0.006	6QG0_G
Structure of eIF2B-eIF2 (phosphorylated at Ser51) complex (model 1) [Saccharomyces cerevisiae S288C]	39.9	0.006	6QG0_H
Structure of eIF2B-eIF2 (phosphorylated at Ser51) complex (model 2) [Saccharomyces cerevisiae S288C]	39.9	0.006	6QG1_G
Structure of eIF2B-eIF2 (phosphorylated at Ser51) complex (model 2) [Saccharomyces cerevisiae S288C]	39.9	0.006	6QG1_H
Structure of eIF2B-eIF2 (phosphorylated at Ser51) complex (model A) [Saccharomyces cerevisiae S288C]	39.9	0.006	6QG2_G
Structure of eIF2B-eIF2 (phosphorylated at Ser51) complex (model A) [Saccharomyces cerevisiae S288C]	39.9	0.006	6QG2_H
Structure of eIF2B-eIF2 (phosphorylated at Ser51) complex (model B) [Saccharomyces cerevisiae S288C]	39.9	0.006	6QG3_G
Structure of eIF2B-eIF2 (phosphorylated at Ser51) complex (model B) [Saccharomyces cerevisiae S288C]	39.9	0.006	6QG3_H
Saccharomyces cerevisiae (baker's yeast) [budding yeasts]			
eIF2B:eIF2 complex [Saccharomyces cerevisiae]	39.9	0.006	6I7T_C
eIF2B:eIF2 complex [Saccharomyces cerevisiae]	39.9	0.006	6I7T_D
Structure of eIF2B-eIF2 (phosphorylated at Ser51) complex (model C) [Saccharomyces cerevisiae]	39.9	0.006	6QG5_G
Structure of eIF2B-eIF2 (phosphorylated at Ser51) complex (model C) [Saccharomyces cerevisiae]	39.9	0.006	6QG5_H
Structure of eIF2B-eIF2 (phosphorylated at Ser51) complex (model D) [Saccharomyces cerevisiae]	39.9	0.006	6QG6_G
Structure of eIF2B-eIF2 (phosphorylated at Ser51) complex (model D) [Saccharomyces cerevisiae]	39.9	0.006	6QG6_H
Ebola virus - Mayinga, Zaire, 1976 [viruses]			
EBOV GP in complex with variable Fab domains of IgGs c2G4 and c13C6 [Ebola virus - Mayinga, Zaire, 1976]	38.3	0.019	5KEL_A
EBOV GP in complex with variable Fab domains of IgGs c2G4 and c13C6 [Ebola virus - Mayinga, Zaire, 1976]	38.3	0.019	5KEL_E
EBOV GP in complex with variable Fab domains of IgGs c2G4 and c13C6 [Ebola virus - Mayinga, Zaire, 1976]	38.3	0.019	5KEL_F
Centruroides sculpturatus (bark scorpion) [scorpions]			
PROTON NUCLEAR MAGNETIC RESONANCE AND DISTANCE GEOMETRY(SLASH)SIMULATED ANNEALING STUDIES ON THE VARIANT-1 NEUROTOXIN FROM THE NEW WORLD SCORPION CENTRUROIDES SCULPTURATUS EWING [Centruroides sculpturatus]	34.5	0.027	1VNA_A
PROTON NUCLEAR MAGNETIC RESONANCE AND DISTANCE GEOMETRY(SLASH)SIMULATED ANNEALING STUDIES ON THE VARIANT-1 NEUROTOXIN FROM THE NEW WORLD SCORPION CENTRUROIDES SCULPTURATUS EWING [Centruroides sculpturatus]	34.5	0.027	1VNB_A
Escherichia virus T4 [viruses]			
Bacteriophage T4 isometric capsid [Escherichia virus T4]	37.6	0.028	5VF3_Z
Komagataella pastoris [budding yeasts]			
Structure of Tra1 subunit within the chromatin modifying complex SAGA [Komagataella pastoris]	35.6	0.17	5OEJ_B
Komagataella phaffii GS115 [budding yeasts]			
Structure of SAGA bound to TBP [Komagataella phaffii GS115]	35.6	0.17	6TB4_L
Structure of SAGA bound to TBP, including Spt8 and DUB [Komagataella phaffii GS115]	35.6	0.17	6TBM_L
Mus musculus (house mouse) [rodents]			
Three-Dimensional Structure Of a Single Chain Fv Fragment Complexed With The peptide GCN4(7P-14P). [Mus musculus]	32.6	0.42	1P4B_L
Crystal Structure of scFv against peptide GCN4 [Mus musculus]	32.6	0.42	1P4I_L
Crystal structure of glycopeptide 22 in complex with scFv-SM3 [Mus musculus]	33.3	0.44	5FXC_H
Understanding the singular conformational landscape of the Tn antigens: Sulfur-for- oxygen substitution in the glycosidic linkage provides new insights into molecular recognition by an antibody [Mus musculus]	33.3	0.44	5N7B_H
Crystal structure of glycopeptide 'GVTSAfPDT*RPAP' in complex with scFv-SM3 [Mus musculus]	33.3	0.44	5OWP_H
Crystal structure of scFv-SM3 in complex with APD-SeThrGalNAc-RP [Mus musculus]	33.3	0.44	6FRJ_H
Crystal structure of scFv-SM3 in complex with compound 3 [Mus musculus]	33.3	0.44	6FZQ_H
Crystal structure of scFv-SM3 in complex with compound 2 [Mus musculus]	33.3	0.44	6FZR_H
scFv-1SM3 in complex with glycopeptide containing an sp2-imino sugar [Mus musculus]	33.3	0.44	6TGG_H
Application of anti-helix antibodies in protein structure determination (9014-1P4B) [Mus musculus]	32.2	0.48	6K65_L
Crystal structure of scFv-SM3 in complex with APD-SGalNAc-RP [Mus musculus]	32.9	0.60	5A2I_H
Crystal structure of scFv-SM3 in complex with the naked peptide APDTRP [Mus musculus]	32.9	0.60	5A2J_H
Crystal structure of scFv-SM3 in complex with APD-TGalNAc-RP [Mus musculus]	32.9	0.60	5A2K_H
Crystal structure of scFv-SM3 in complex with APD-CGalNAc-RP [Mus musculus]	32.9	0.60	5A2L_H
Candida albicans SC5314 [budding yeasts]			
Candida albicans PKh Kinase Domain [Candida albicans SC5314]	32.6	1.1	4C0T_A

Staphylococcus aureus subsp. aureus NCTC 8325 [firmicutes]				
**Crystal structure of the ligand binding region of staphylococcal adhesion SraP [Staphylococcus aureus subsp. aureus NCTC 8325]**	30.3	6.0		**4M00_A**

Taxonomy

Taxonomy	Number of hits	Number of Organisms	Description
root	71	11	
. cellular organisms	67	9	
.. Opisthokonta	66	8	
... Bilateria	46	3	
.... Euarchontoglires	44	2	
..... Homo sapiens	30	1	**Homo sapiens hits**
..... Mus musculus	14	1	**Mus musculus hits**
.... Centruroides sculpturatus	2	1	**Centruroides sculpturatus hits**
... Saccharomycetales	20	5	
.... Saccharomyces	16	2	
..... Saccharomyces cerevisiae	6	2	**Saccharomyces cerevisiae hits**
...... Saccharomyces cerevisiae S288C	10	1	**Saccharomyces cerevisiae S288C hits**
.... Komagataella	3	2	
..... Komagataella pastoris	1	1	**Komagataella pastoris hits**
..... Komagataella phaffii GS115	2	1	**Komagataella phaffii GS115 hits**
.... Candida albicans SC5314	1	1	**Candida albicans SC5314 hits**
.. Staphylococcus aureus subsp. aureus NCTC 8325	1	1	**Staphylococcus aureus subsp. aureus NCTC 8325 hits**
. Viruses	4	2	
.. Ebola virus - Mayinga, Zaire, 1976	3	1	**Ebola virus - Mayinga, Zaire, 1976 hits**
.. Escherichia virus T4	1	1	**Escherichia virus T4 hits**

Top
